# Quantitative TGA distinguishing physisorbed and chemisorbed water in the ligand layer of iron oxide nanoparticles

**DOI:** 10.1039/d6na00573j

**Published:** 2026-08-28

**Authors:** Mirijam Zobel, Maksim Plekhanov, Sascha Ehlert, Andreas Magerl

**Affiliations:** a RWTH Aachen University, Institute of Crystallography Jägerstrasse 17-19 52066 Aachen Germany zobel@ifk.rwth-aachen.de; b Forschungszentrum Jülich GmbH, JCNS-3: Neutron Analytics for Energy Research Wilhelm-Johnen-Straße 52428 Jülich Germany; c Forschungszentrum Jülich GmbH, JCNS-1: Neutron Scattering and Soft Matter Wilhelm-Johnen-Straße 52428 Jülich Germany; d Friedrich-Alexander Universität, Biophysics Group, Department of Physics Henkestraße 91 91052 Erlangen Germany

## Abstract

The details of the interfacial hydration of ligand-grafted nanoparticle surfaces depend on the chemistry and grafting density of the ligand molecules. Superparamagnetic iron oxide nanoparticles (IONPs) are used in ferrofluids and biomedicine, for example, where surface functionalization with citrate is of particular interest. Despite recent progress in quantifying citrate content, identifying and enumerating interfacial water remains challenging. In this study, we examine IONPs with a diameter of 7.4 ± 1.1 nm stabilised with either citrate or diethylene glycol with a coverage ranging from a monolayer up to 8 wt% ligand content. Thermogravimetric analysis (TGA) of IONP powders equilibrated at 30 and 75% relative humidity can be fitted consistently with two models: either the activation energy requires a distribution reflecting heterogeneity in sorption sites, or a reduction of the surface area available for evaporation near the end of the drying process. In either case, two distinct water species are identified with desorption energies of 29–33 kJ mol^−1^ and of 40–46 kJ mol^−1^, characteristic of physisorbed and chemisorbed water, respectively. In this case, chemisorbed water does not involve dissociation but rather consists of water which is hydrogen-bonded to functional groups of the ligand molecules. Vapour sorption isotherms confirm the TGA data, which were collected at a rate of 5 K min^−1^ to resolve the interfacial water species. Thus, widely available TGA experiments can provide quantitative relationships between surface chemistry and hydration behaviour.

## Abstract 2

Thermogravimetric experiments distinguish water molecules chemisorbed to citrate ligands and physisorbed water. The concept of cluster-to-film adsorption describes both TGA and vapor sorption data.

## Graphical abstract

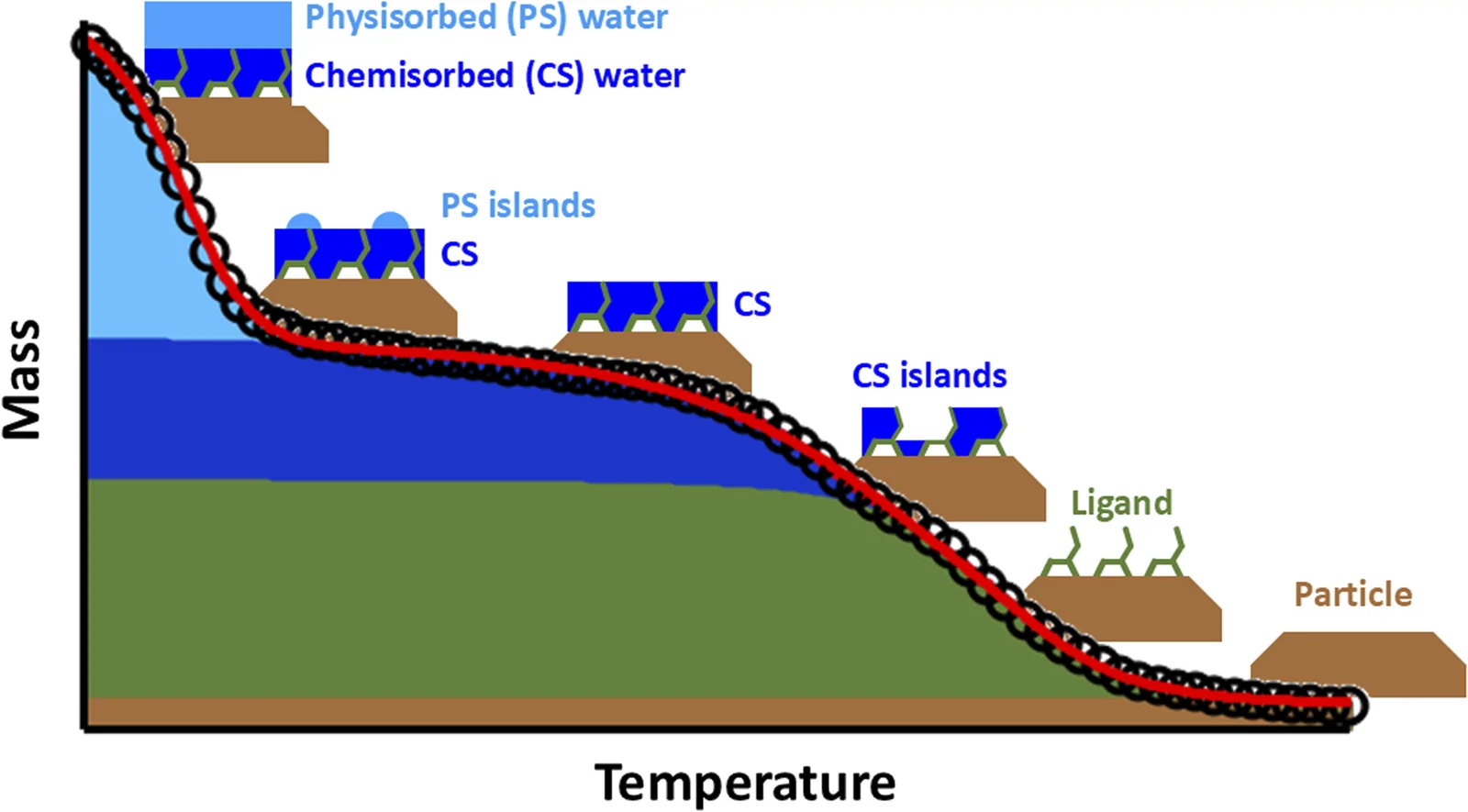

## 1. Introduction

In applications for drug delivery, magnetic resonance imaging or catalytic reactions, iron oxide nanoparticles (IONPs) are typically stabilized with organic ligands and they are often found in aqueous dispersions or in contact with a humid atmosphere. Citrate (CIT) is a biologically non-hazardous ligand, which provides good stabilization in aqueous dispersion and is frequently employed for colloidal stabilization of IONPs.^1^ The interaction of water with the IONP surfaces and the ligand corona is a crucial factor for an understanding of interfacial processes. It is common grounds that storing nanoparticle (NP) powders in humid atmospheres or suspension can have detrimental effects including secondary NP growth^2^ and phase transformations,^3^ in particular for non-stabilized NPs.

Water sorption and corresponding heat of adsorption on bare iron oxide surfaces or IONP powders have been intensely studied experimentally in the past involving amongst others vapor sorption isotherms supported by computer simulation,^4^ calorimetric measurements and inelastic neutron scattering.^5^ In a first step, dissociative adsorption forms a hydroxyl covered IONP surface, followed by multilayer physisorption at higher vapor pressures and culminating in capillary condensation between the NPs. In order to model corresponding water sorption isotherms of beds of NP powders including vapor pressures of *p*/*p*_0_ > 0.8, the GAB equation, established by Guggenheim, Andreson and De Boer for up to *p*/*p*_0_ = 0.8, has been extended by Ribeyre *et al.* to include the effect of capillary condensation in the space between the particles based on Kelvin's law.^6^ Hereby, *p*/*p*_0_ refers to the ratio of the applied to the saturation pressure of water vapor at a given temperature.

The solid quantification of CIT coverage on IONPs is ideally a multi-method undertaking. Since thermogravimetric analysis (TGA) can only access the total weight loss or respective carbon-related weight loss when coupled to mass spectrometry (MS), further element-specific techniques such as elemental (CHNOS) analysis or ligand-selective techniques like chromatography or UV detection are required to separate carbon content belonging to CIT from carbon in residual solvent molecules.^7,8^

In order to investigate the physicochemical properties of water molecules in the interfacial layers of CIT-covered IONPs, we additionally need to identify the water content across a large range of relative humidities. The quantification of the water content is particularly important when employing hydrogen-sensitive techniques such as neutron spectroscopy to investigate the geometry and energetics of diffusion dynamics of water and ligand molecules on the surface of metal oxide nanoparticles.^9^

Usually in TGA analysis, the first mass loss is attributed to the total amount of adsorbed water^8^ without uncovering information about the distinct binding modes of interfacial water species which could be extracted though from the time and/or temperature dependence of the mass loss. Here, we address this limitation by considering a quantitative analysis of TGA data coupled with MS, cross-validating our findings with water vapor sorption experiments.

## 2. Experimental details

### 2.1 Sample synthesis

The polyol synthesis of the IONPs follows our previous work^7^ which follows earlier synthesis approaches in diethylene glycol (DEG) solvent with subsequent ligand exchange with ligands containing carboxylic acid groups:^10,11^ a solution of 16 mmol NaOH (≥99.0%, Merck) in 40 g of DEG (99.0%, Merck) (*c*_NaOH_ = 0.22 mol L^−1^) is added under stirring to a precursor solution containing 2 mmol FeCl_2_·4H_2_O (99.0%, Thermo Fisher) and 4 mmol FeCl_3_·6H_2_O (>97.0%, Thermo Fisher) in 80 g of DEG (*C*_FeCl_3_+FeCl_2__ = 0.17 mol L^−1^). The reaction solution is heated in a 250 mL flask from room temperature (RT) to 493 K with a ramp of 130 K h^−1^ under N_2_ flow (grade 5.0), where it is kept for one hour. Subsequent free cooling to RT takes about two hours. The solvent DEG acts as a weak stabilizer to the IONPs. To replace the DEG by the stronger binding CIT ligand, 2.5 mmol trisodium citrate dihydrate (98.0%, Thermo Fisher) dissolved at RT in a mixture of 5 mL of H_2_O and 5 mL of DEG (*C*_citrate_ = 0.25 mol L^−1^) are added to the synthesis mixture during the cooldown at 403 K. For both ligands 200 mL of acetone (≥99.0%, Merck) is added at RT to precipitate the dispersed IONPs. Afterwards, the IONP precipitate is washed with absolute ethanol (≥97.0%, Thermo Fisher) several times using 150 mL of ethanol per washing cycle. After each cycle, IONPs were precipitated using a magnet placed underneath a beaker. Excess solvent is discarded, and the powders are dried at RT for 24 hours under lab conditions and stored as dried powders. The stability of our IONP powders including particle sizes and crystallinity over several months has been demonstrated before, even for IONPs as small as 5 nm in diameter.^7^

To reduce the ligand grafting density (GD), IONPs are subjected to additional washing (Table S1). In the case of CIT, water washing is performed for up to six cycles, adding 50 mL of Milli-Q water in each cycle, followed by washing with 100 mL of ethanol. DEG GD is reduced using ethanol instead of Milli-Q water.

To establish the baseline GD, dialysis was conducted for up to ten days under continuous stirring with daily exchange of dialysate water. The dispersed IONPs (20 g L^−1^) were filled into a dialysis tube (cellulose hydrate tubes, Nadir, Germany), sealed, and placed in a 2.5 L beaker on a magnetic stirring plate. The beaker was filled with water and with ethanol for CIT- and DEG-capped IONPs, respectively. The pH of dispersed powders was measured with a pH-meter Metrohm 744 (Metrohm, Switzerland).

### 2.2 Water sorption measurements

Water vapor sorption isotherms were measured static-volumetrically in a 3P vapor 200 (3P Instruments, Germany) at 303 K. Before each measurement, powders were exposed for four hours to a vacuum of 10^−3^ mbar. Subsequently, helium was dosed to determine the free volume in the sample chamber. As a next step, a specific amount of water vapor is introduced, and the adsorbed fraction is calculated from the pressure drop. The adsorbed volume of water was converted into a weight fraction – *ω*_H_2_O_ in wt% using1
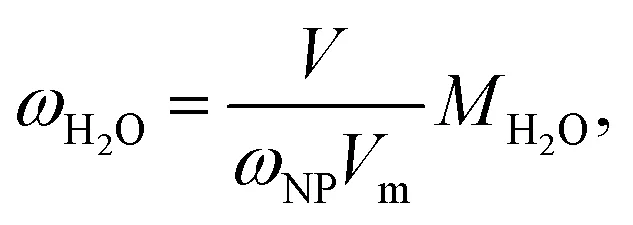
where *V* is the adsorbed volume of water vapor per gram of bare NPs, *V*_m_ is the molar volume of gas, *ω*_NP_ is the weight fraction of IONPs with the ligand, but without water, obtained from TGA and *M*_H_2_O_ is the molar mass of H_2_O.

As controlled by TGA analysis before and after water sorption experiments, the ligand content for both CIT and DEG samples remains unchanged by water sorption measurements and evacuation, as shown in Fig. S5.

### 2.3 Thermogravimetric analysis

From TGA measurements carried out in the 303–1173 K temperature range in N_2_ flow on a Jupiter STA 449 F3 (NETZSCH, Germany) with a heating ramp of 5 K min^−1^ we determined the amount of ligand and water in the IONP powders. The mass loss observed before a CO_2_ signal is recorded in the exhaust gas measured with the quadrupole MS HPR-20 EGA (Hiden Analytical, UK) is attributed to the evaporation of water (see Fig. S7). This mass loss of water can be further subdivided into chemisorbed (CS) and physisorbed (PS) water by fitting of the TGA line shape, see below Section 3.4). The mass loss above the onset of the CO_2_ signal stems from the ligand, and the remaining mass at very high temperatures was assigned to iron oxide, see Fig. S5. We had previously made the effort to quantify and distinguish CIT content from possible residual solvents by combining TGA, CHN analysis and IR.^7^ Based on this and given the washing procedure, we ascertained that no residual DEG solvent is contained in CIT samples. It is important to note our heating ramp of only 5 K min^−1^, while usually 10 K min^−1^ are used in most investigations.^12,13^ Higher heating ramps may prevent a proper resolution to uniquely decompose the individual desorption or decomposition steps.

### 2.4 Fourier transform infrared spectroscopy

IR spectra in the range of 400–4000 cm^−1^ were recorded with an FT/IR-4X spectrometer (Jasco, Germany) in the attenuated total reflectance (ATR) mode with a resolution of 1 cm^−1^. Deconvolution of IR spectra after background subtraction was performed by fitting a sum of Gaussian functions. A Gaussian with the wavenumber *ν* as the variable is defined by2
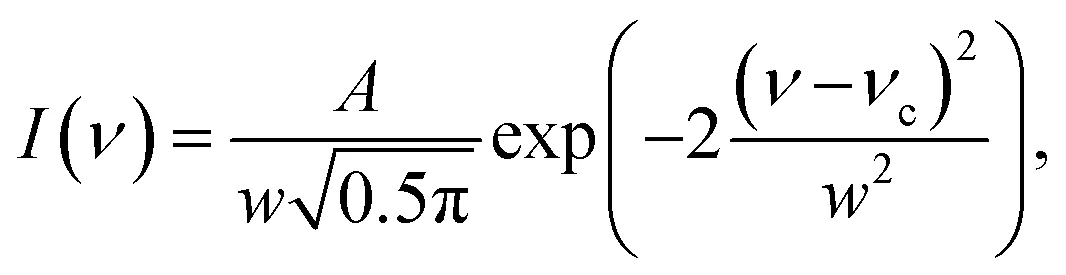
with *I*(*ν*) representing the intensity, *A* the integrated area, *w* the Gaussian width, and *ν*_c_ the peak center. Furthermore, a constant background has been considered in the fit. The deconvolution was performed in the wavenumber range of 1110–1790 cm^−1^, necessitating four Gaussian functions.

### 2.5 Powder X-ray diffraction

Powder X-ray diffraction (PXRD) patterns were collected on dry powders at the Institute of Energy Technologies (IET-1, Forschungszentrum Jülich) with an Empyrean diffractometer (Panalytical, Netherlands) with Mo Kα radiation (*λ* = 0.709 Å) in the momentum transfer range *Q* between 0.7 and 8.2 Å^−1^. The empty sample container measurement was subtracted from the data. The instrumental resolution was considered by refining a LaB_6_ standard data set and structure refinements were performed by the Rietveld method using GSAS-II software, see the SI for further details.^14^

### 2.6 Dynamical light scattering

Dynamical light scattering (DLS) measurements were carried out on a Litesizer 500 (Anton Paar, Austria) in backscattering mode with a wavelength of 660 nm. Powders were dispersed in water with a concentration of 1 g L^−1^ and measured at 298 K. Solvent viscosity was set to 0.8511 mPa s^−1^, the refractive index of the solvent and IONP to 1.3301 and 2.3600, respectively. Number-weighted particle size distributions are displayed in Fig. S1.

### 2.7 Transmission electron microscopy

Transmission electron microscopy (TEM) images were taken with a JEOL-F200. The standard procedure for sample preparation includes partial drying of a diluted IONP solution in water on a carbon-coated copper grid, followed by the removal of excess solution. Histograms of particle size distributions were obtained with ImageJ software. At least 100 measurements of diameter were performed per sample to produce the histograms. In some cases when particle agglomerates were observed, individual IONPs were visually distinguished and evaluated individually, see histograms of counted IONPs in Fig. S2 with the fitted lognormal particle size distribution.

## 3. Results and discussion

### 3.1 Particle size

The size of the IONPs of all samples investigated within this study is 8 nm within errors, as determined by DLS for all samples and selectively validated with PXRD and TEM (Table S1). The size difference observed between the three techniques relate to the physical properties measured: DLS provides the number-weighted hydrodynamic diameter *D*_DLS_ (as converted from intensity distribution), including surface ligands and hydration shell, TEM reveals the NP core size *D*_TEM_, while PXRD determines the coherence length of crystalline domains *D*_PXRD_ within the NP core. In consequence, it can be expected that *D*_DLS_ > *D*_TEM_ > *D*_PXRD_. See SI for further details.

### 3.2 Ligand grafting density and surface coordination

The replacement of DEG by CIT has been demonstrated previously.^7^ The ligand GD of this study was calculated from the mass loss in TGA for temperatures above the water desorption (see Section 3.4 and Fig. S5 and 6) and from the size of the IONPs (see SI Section 1.2 for the calculation). A regular truncated octahedral shape as suggested by TEM (see Fig. S2) was used, although the IONPs may be of a more irregular shape, which may increase the error margins to some extent. In the following, sample names are composed of the abbreviated ligand name CIT or DEG and the ligand GD in molecules per nm^2^, separated by “−”. Various densities of CIT and DEG ligand coverages were prepared by differing numbers of washing cycles, see Table S1.

#### 3.2.1 Diethyleneglycol

**Fig. 1:**
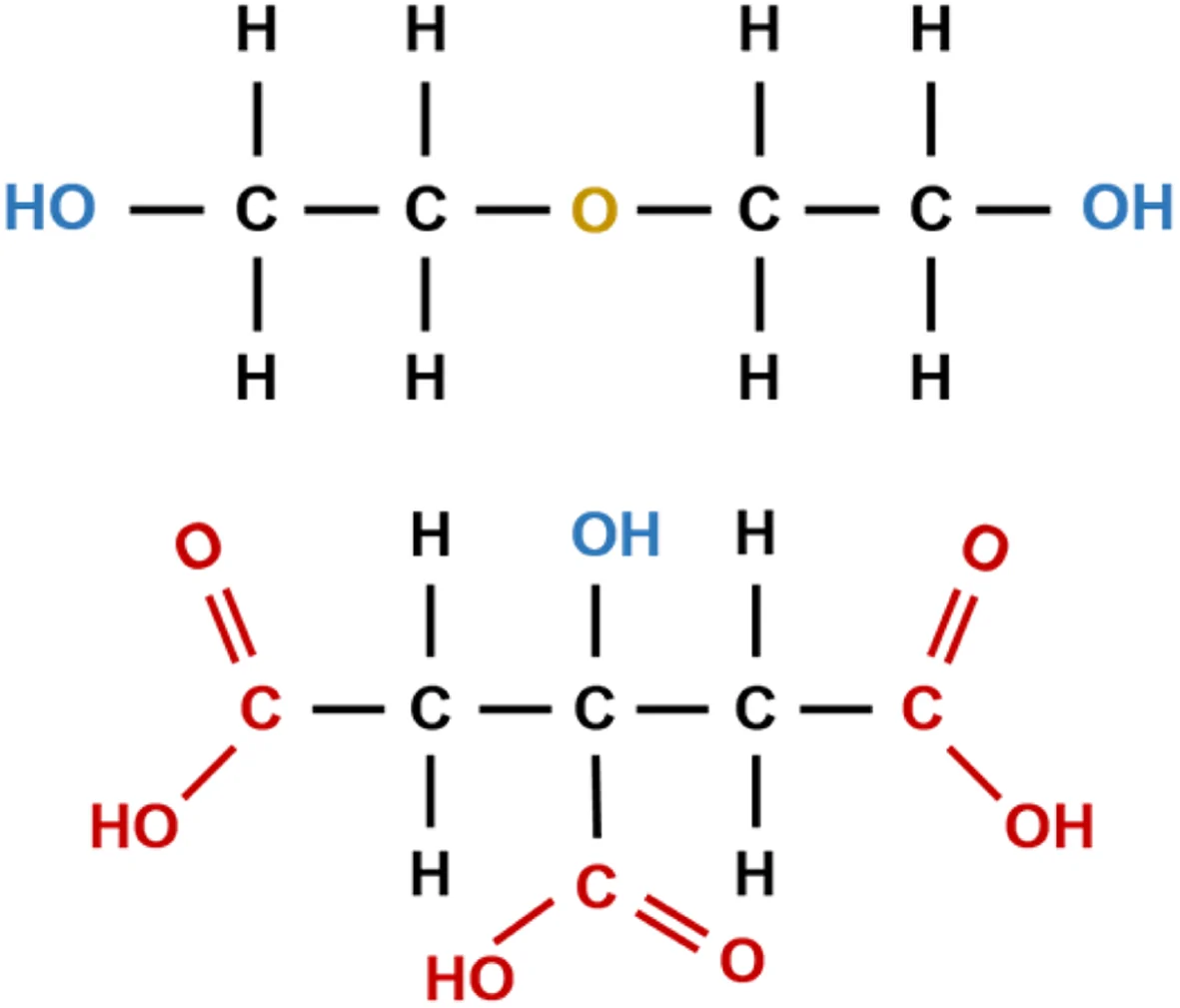
Schematics of the DEG, C_4_H_10_O_3_ (top), and the CIT, C_6_H_8_O_7_, molecule (bottom), highlighting the functional groups: carboxyl in red, hydroxyl in blue, ether oxygen in yellow.

The small, flexible and symmetric DEG molecule C_4_H_10_O_3_ consists of a linear ether backbone with two terminal hydroxyl groups and exists in different conformers in liquid and solid phases^15^ (see also Fig. 1). DEG coordinates weakly only with electrostatic interactions *via* its hydroxyl groups to the IONP surface and thus is readily replaced during synthesis by CIT with its strongly binding carboxyl (COOH) groups. In molecular dynamics (MD) simulations on the sorption behaviour of DEG molecules onto surfaces of (10–14) calcite and (0001) hematite from 50 : 50 solutions of DEG : water, Olsen *et al.* found that DEG molecules bind either with one or with both of the hydroxyl groups to both surfaces.^16^ For triethylene gylcol (TEG) on calcite, they found TEG to bind with both hydroxyl groups to two adjacent oxygen sites for low TEG concentrations. For increasing TEG concentrations, only one hydroxyl group binds and the molecule is oriented perpendicular to the surface.^17^

The lowest DEG GD we observed was 2.1 DEG per nm^2^, similar to the minimal CIT GD. This lowest GD was achieved by sequential washing cycles. Geometrically, we expect that the DEG molecules bind with both hydroxyl groups to the surface. For higher DEG GDs of 3.2 and 4.9 DEG per nm^2^, it is not evident whether the lower value can still be explained with a monolayer of upright DEG molecules bound *via* one hydroxyl group and at which GD a multilayer formation sets in. The measured *D*_DLS_ values (Table S1) including the range of the ligand and hydration shell are not precise enough to derive the difference in parallel and upright DEG orientation.

Water interacts with DEG molecules *via* the hydrogen atoms of water either forming hydrogen bonds with the hydroxyl oxygens or with the oxygen of the central ether group of the DEG molecule.^18^

#### 3.2.2 Citrate

The structure of the CIT molecule indicated in Fig. 1 features three carboxyl and a central hydroxyl group, which allows for different binding configurations to the IONP surface (see Fig. S4 for a sketch): in monodentate binding only one oxygen of the COO^−^ group binds to the surface; in bidentate binding, both oxygens of a COO^−^ group bind to the same iron atom, and in bridging mode two oxygens of the same COO^−^ group bind to two different iron atoms.^19,20^ The details of the coordination behavior depend on numerous factors, including pH, surface termination, crystalline facet exposed, and ligand grafting density. Zhang *et al.* provide a rather complete overview of these binding options at low and high pH values, see Scheme 1 therein,^21^ which differ in the involvement of the hydroxyl group in the surface binding for the alkaline case (be aware that pH is measured in solution, FT/IR typically in the powder and NP interfaces can feature interfacial pH values differing from the bulk solution pH significantly).

FT/IR measurements of our CIT-covered IONP powders for GD ranging from 2.3 to 5.7 and subsequent deconvolution of the spectra (see Fig. S4 and Table S3) show that surface stabilization is induced by binding of the carboxylic groups to surface atoms. Previous X-ray Absorption Spectroscopy (XAS) analysis of our CIT-covered IONPs revealed the absence of surface hydroxyl groups.^22^ The occurrence of the C–OH stretching band of CIT at ∼1280 cm^−1^ further indicates that the central hydroxyl group of CIT does not take part in the surface coordination (see peak IV in Fig. S4). For increasing CIT GD, the binding mode transitions from monodentate for CIT-2.3 to bidentate and bridging for CIT-5.7. The sample CIT-5.7 corresponds to having about 2.5 CIT layers. In such a scenario, CIT molecules beyond the monolayer coverage form hydrogen bonds to the CIT molecules in the inner monolayer, which is again in line with the multilayer BET adsorption model of Zhang *et al.*, in which CIT molecules beyond the monolayer coverage bind as outer-sphere complexes; a similar behaviour was found for CIT multilayers on hematite.^23^ Alternatively, the strong chelation effect of CIT with unreacted Fe^2+^ or Fe^3+^ could form additional iron-citrate complexes, which co-exist as a side-phase, which in small amounts and amorphous in nature would not be detected in the PXRD. Such chelate complexes are however not discussed or observed in previous investigations.^19,21^

**Fig. 2:**
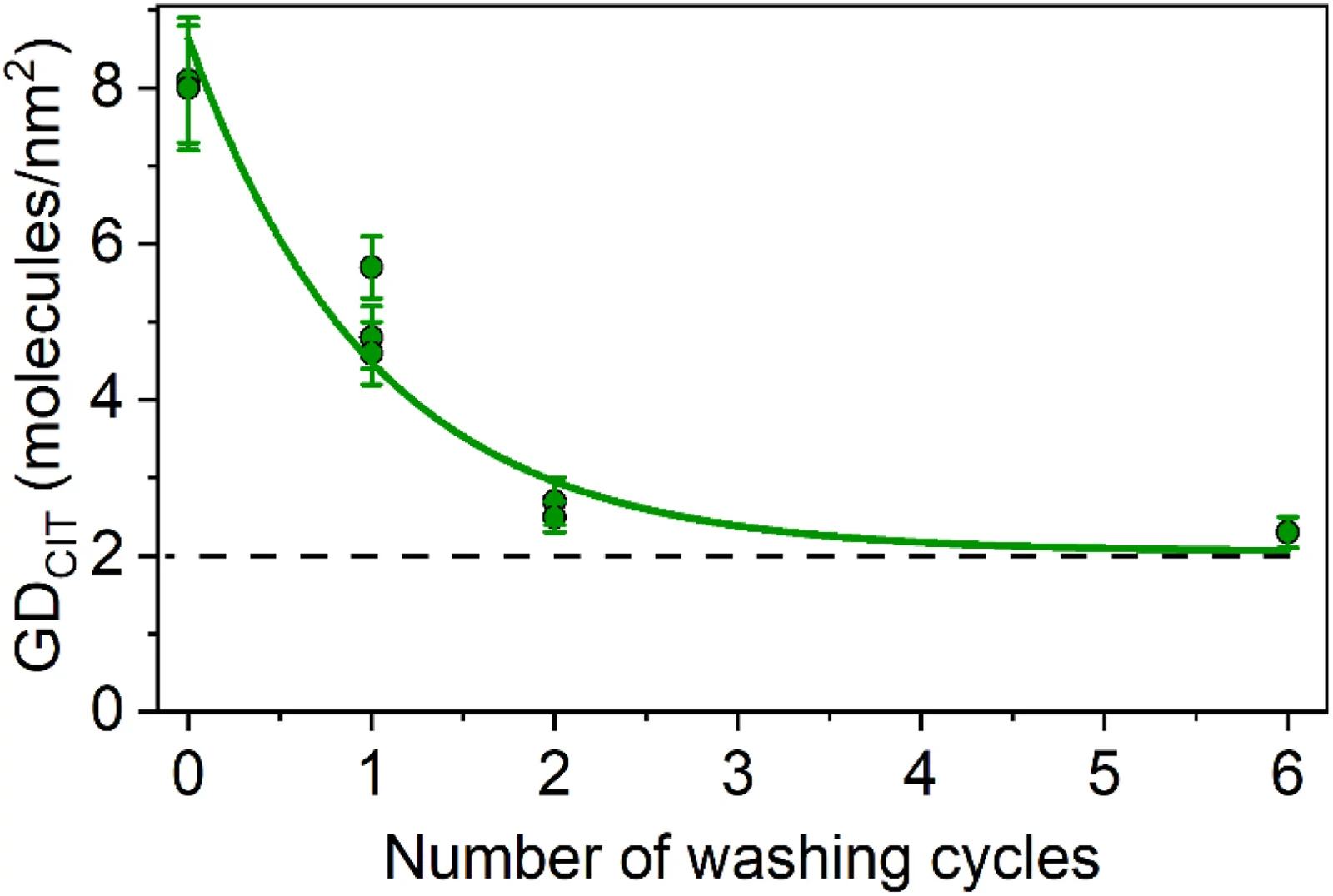
GD for CIT-covered IONPs as a function of water washing cycles. Green curve represents exponential fit.

Fig. 2 shows that the GD of CIT drops after washing with water from an initial value after synthesis of 8.0 CIT per nm^2^ approximately exponentially to a limiting value of 2.1 ± 0.2 nm^2^ for a high number of washing cycles with an experimental value of 2.3 ± 0.2 CIT per nm^2^ for 6 washing cycles. This lower limit of the CIT GD was further confirmed by dialysis. Ten days of dialysis with daily exchange of dialysate water resulted in the sample CIT-2.3 with 2.3 ± 0.2 molecules per nm^2^. Hence, this fraction of CIT molecules binds strongly to the IONP surface in aqueous dispersion forming a monolayer with a GD of 2.3 CIT per nm^2^. This monolayer coverage matches well previous findings of a 2.3 CIT per nm^2^ monolayer coverage established in a detailed attenuated total reflection FT/IR investigation on magnetite NPs of 11 nm in diameter.^21,23^

### 3.3 Water vapor sorption on ligand-grafted IONPs

Water sorption processes on bare IONPs without ligands have been investigated both experimentally and computationally.^4,14^ Yet, the influence of surface ligands on water adsorption has not been studied through measurement of water sorption isotherms, as to our knowledge. Here, we report such measurements for DEG- and CIT-covered IONPs, allowing for a direct comparison of their interfacial hydration behaviours.

#### 3.3.1 Impact of ligand type and grafting density

**Fig. 3:**
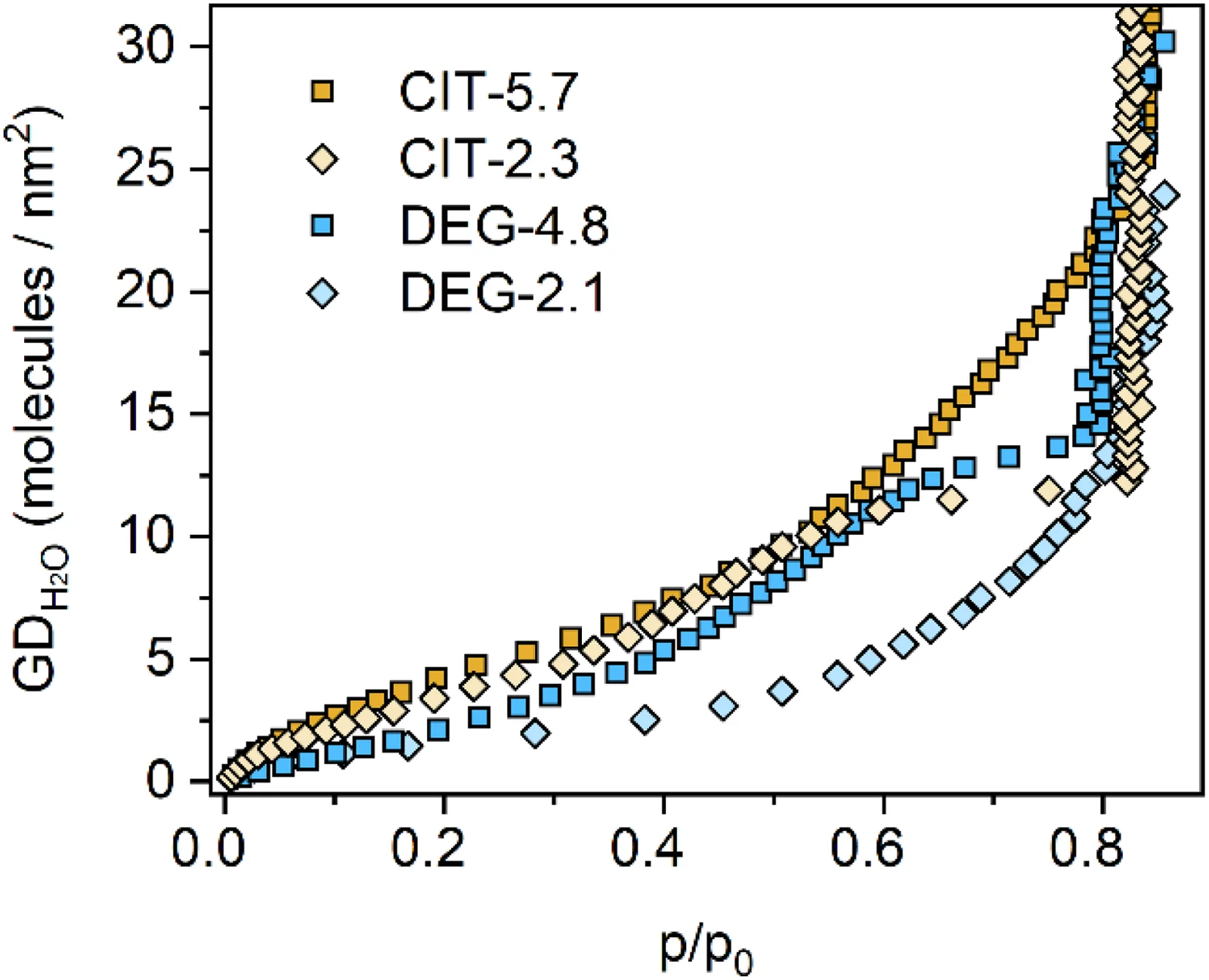
Adsorption isotherms of CIT- and DEG-covered IONPs at 303 K. The *p*/*p*_0_ is the ratio of the applied to the saturation pressure of water vapor at the loading temperature.

Fig. 3 shows the water sorption isotherms for IONPs covered with two different GDs of CIT and DEG each. The GD_H_2_O_ of adsorbed water molecules per nm^2^ is calculated using:3
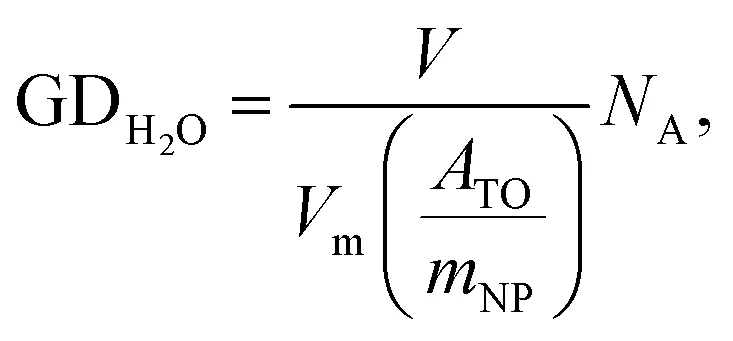
where *V* is the measured volume of water vapor adsorbed per gram of bare NPs in m^3^ g^−1^, *V*_m_ is the molar volume of water vapor in m^3^ mol^−1^, *A*_TO_ is the surface area of a single NP in m^2^, *m*_NP_ is the mass of a single NP, and *N*_A_ is the Avogadro number in mol^−1^ (see SI, Section 1.1 for calculation of *A*_TO_ and *m*_NP_).

At low pressures the absorption isotherms show a type II Langmuir behaviour according to the IUPAC sorption isotherm classification with convex regions to the pressure axis indicative of the progressive filling of a specific first absorption site. While this layer is normally attributed to a first surface layer, we note that this classification may be misleading in the present context because of the structural complexity of the surface of coated IONPs. The adsorption behaviour changes character with inflection points at about *p*/*p*_0_ < 0.1 for both ligands marking the onset of competing adsorption sites. The water uptakes at the inflection point are higher by about a factor of 2 for CIT as compared to DEG which may relate to the large number of carboxyl groups in CIT as strong anchoring sites for CB water molecules, while the GD of ligands seems to be of minor importance.

At higher vapor pressure, the water uptake becomes sensitive to the ligand type and GD. Bulk-like condensation occurs in the interparticle space of the powder in all cases for 0.8 < *p*/*p*_0_ < 0.9. For CIT-2.3, the adsorption isotherm takes on a type I behaviour signifying saturation of anchoring sites, while for CIT-5.7, the type II behaviour is maintained up to condensation, indicative of weak interactions between the adsorbate and the adsorbent leading to a structureless formation of multilayers. In the case of the DEG-2.1 sample, the water uptake is low with an adsorption curve type II, while the DEG-4.8 sample shows a transition to type I with a saturation of a well-defined layered adsorbate until bulk-like condensation sets in.

We note as a common feature of both ligands that the water uptake increases with the ligand GD as a larger number of ligand molecules augments the number of unbound carboxyl groups in the case of CIT or ether- and hydroxyl groups in the case of DEG and thus more docking sites for water molecules are being offered (see Fig. 3).

#### 3.3.2 Hysteresis of vapor sorption curves on CIT-covered IONPs

**Fig. 4:**
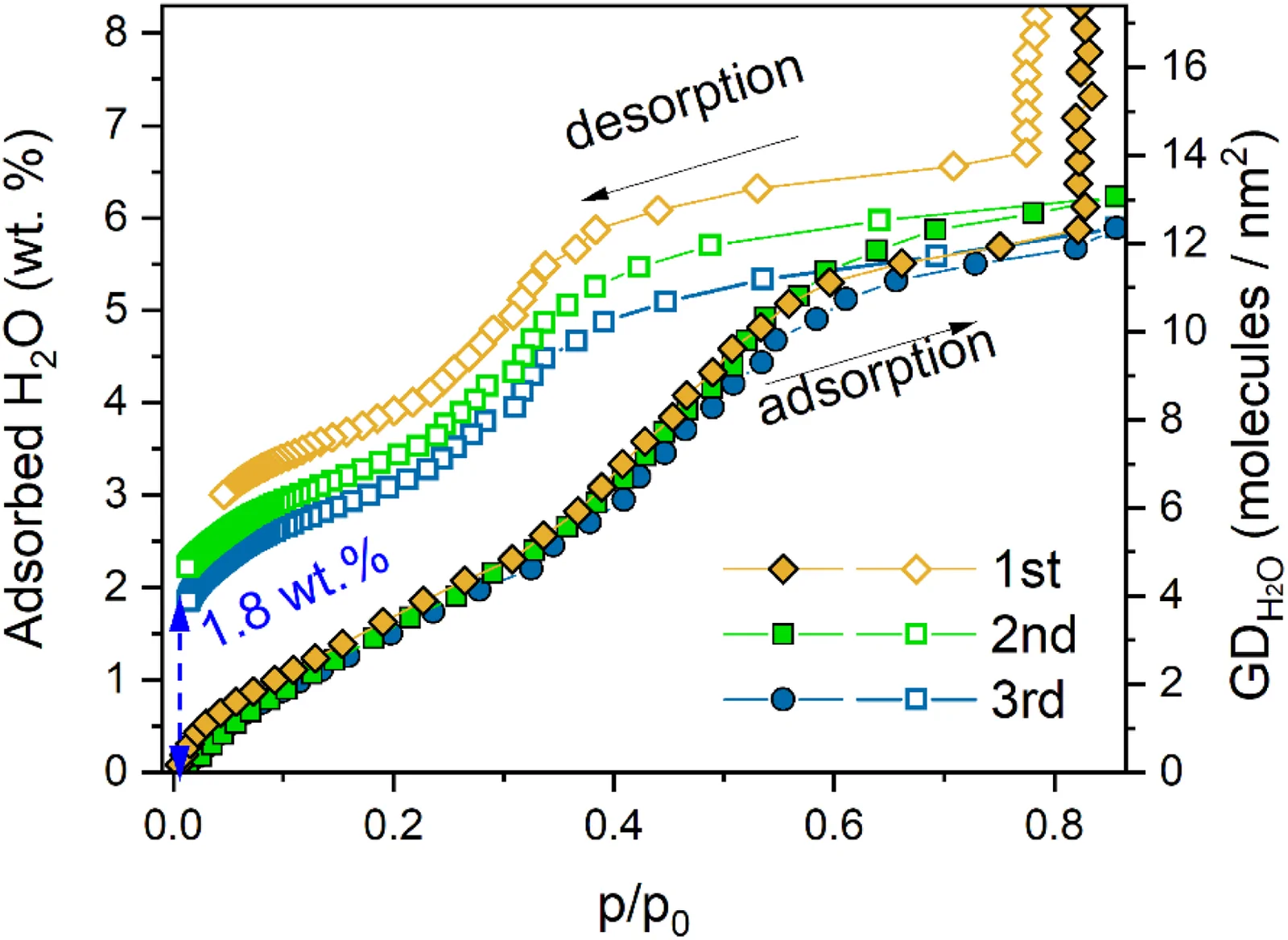
Adsorbed water for three subsequent adsorption and desorption cycles for CIT-2.3. A four-hour evacuation down to a pressure of 10^−3^ mbar between the cycles was needed to reach the same nominal water-free starting condition.

The reversibility and hysteresis of water sorption is shown in Fig. 4 for CIT-2.3 measured in three cycles with the same loading protocol. The sample had been placed under vacuum for four hours down to a pressure of 10^−3^ mbar to reach the same nominal water-free starting condition for all loadings. The amount of retained water at the lowest pressure reachable in the sorption apparatus for the three cycles ranges from 1.8 ± 0.1 wt% to 2.6 ± 0.1 wt% (corresponding to 3.5 ± 0.4 to 5.2 ± 0.5 water molecules per nm^2^), which is close to the 2.7 ± 0.5 wt% of chemisorbed water observed in TGA for the CIT-2.3 sample (see eqn (1) and Table S4).

Viisanen *et al.* have classified hysteresis over bare nonporous mineral surfaces into two types: cluster-wise adsorption with contact angle hysteresis for hydrophobic surfaces and cluster-to-film adsorption for hydrophilic surfaces.^24^ The shape of our hysteresis loops on CIT-grafted iron oxide surfaces resembles the cluster-to-film adsorption, in which the condensation starts in individual patches, which at a later stage converge into a film. In this stage, evaporation during desorption proceeds first by thinning the film. This results in different surface areas and curvatures for water agglomerates on the adsorption and desorption branches at the same RH (see also Section 3.4). The equilibrium vapor pressure over a curved liquid surface is higher than over a flat surface relating to the Kelvin effect.^25^ For a cap-shaped meniscus or droplet, the Kelvin relationship gives the shift of equilibrium vapor pressure *p* relative to the saturated vapor pressure over a flat surface *p*_sat_ as4
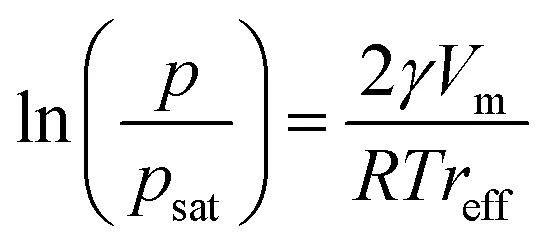
where *γ* is the water surface tension, *V*_m_ the molar volume of water, and *r*_eff_ is the effective radius of curvature of the liquid–vapor interface. In consequence, the transition from a closed adsorbate layer to a droplet/patch structure becomes delayed for desorption as compared to the sorption branch.

According to ref. 26, hysteresis is more pronounced in samples which have seen a high water content. Fig. 4 shows this correlation of the hysteresis width with the amount of water accumulated during adsorption for three subsequent adsorption–desorption runs of CIT-2.3.

Capillary condensation in interparticle space takes place for the 1st adsorption cycle at *p*/*p*_0_ ∼ 0.8, but not for the 2nd and 3rd cycles in the accessed pressure range. This implies that the condensation in the 1st cycle leads to a modification of the IONPs with an altered void structure. This reformed – and presumably reduced – void structure reduces the water uptake in the 2nd and 3rd run, which in turn reduces hysteresis widths. Post-sorption inspection of the sample revealed an apparent consolidation from their initial powdered state to a more compact appearance. However, this morphological change appears to be reversible as the samples could be easily redispersed in water without a change in particle size (*D*_DLS_ of 6.6 ± 1.2 and 6.7 ± 1.2 nm before and after the sorption experiment) suggesting weak interparticle adhesion rather than a permanent structural alteration. In addition, the ligand amount remained unchanged by the water sorption measurements (see Fig. S5).

### 3.4 Distinction of chemi- and physisorbed water *via* modelling of TGA data

Insights into the binding of sorbed water beyond the global amount of water may be obtained from a detailed analysis of the shape of TGA spectra. Sorbed water in the CIT and DEG samples evaporates in two steps, visible by two regimes of mass losses before a significant CO_2_ signal in the MS data sets in, which is evidence for ligand decomposition (see Fig. S7). The MS H_2_O signal recorded in parallel to the CO_2_ signal does not originate from water desorption, but from water released from the ligand decomposition. The different loss steps during ligand decomposition have been correlated before with the different binding configurations of the CIT molecule on the IONP surface.^21^

**Fig. 5:**
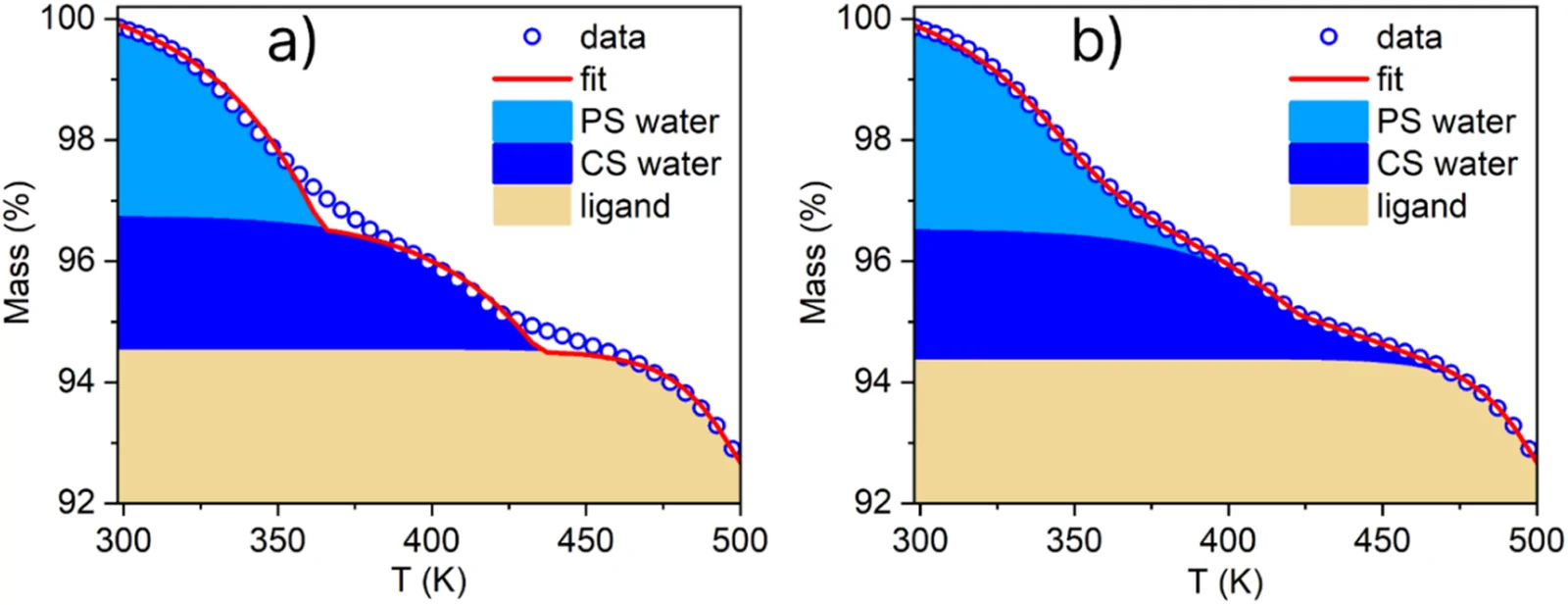
TGA fits for the water desorption for sample CIT-5.7 equilibrated at 30% RH for (a) thermodynamic evaporation only (OEP) according to eqn (5), and (b) the RES model. PS and CS denote physi- and chemisorbed water, respectively.

In the following we present models for a quantitative description of full TGA curves including the ligand decomposition. In an initial approach, the mass reduction *m*_*i*_ as a function of temperature *T* for each process *i* in the TGA measured with the heating rate *β* is described by a thermally activated process characterized by a prefactor *k*_*i*_ and an activation energy of evaporation *E*_*i*_ according to5
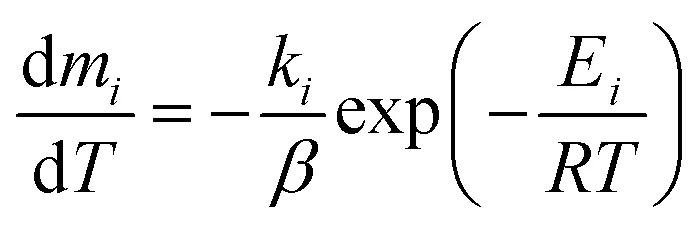
with the universal gas constant *R* = 8.3145 mol^−1^ K^−1^. This description invoking only evaporation processes (OEP) produces shoulders with sharp kinks as the essential features of a TGA curve (Fig. 5a). The fitted thermodynamic parameters for the sample CIT-5.7 at 30% RH are given in Table 1.

**Table 1:** TGA fit parameters for the OEP, RES, and DAE model for the two water desorption peaks for CIT-5.7 at 30% RH. PS and CS denote physi- and chemisorbed water

		OEP	RES	DAE
**1st water desorption peak (PS water)**				
*k*_PS_ × 10^−5^/s^−1^		1.12 ± 0.11	1.16 ± 0.16	1.19 ± 0.25
*E*_PS_/kJ mol^−1^		29.50 ± 0.02	29.51 ± 0.33	29.26 ± 0.36
*σ*_PS_/*i*		—	—	1.14 ± 0.18
*s*_PS_/wt%		3.29 ± 0.13	3.51 ± 0.07	2.65 ± 0.33
*T*_transPS_/K		—	353.0 ± 0.9	—
*G*_PS_/K		—	8.8 ± 0.7	—
	
**2nd water desorption peak (CS water)**				
*k*_CS_ × 10^−5^/s^−1^		7.99 ± 12.11	7.85 ± 0.59	9.39 ± 2.22
*E*_CS_/kJ mol^−1^		42.12 ± 0.33	42.22 ± 0.01	41.72 ± 0.61
*σ*_CS_/*i*		—	—	2.73 ± 0.53
*s*_CS_/wt%		2.19 ± 0.14	2.13 ± 0.12	2.84 ± 0.36
*T*_transCS_/K		—	422.8 ± 1.8	—
*G*_CS_/K		—	0.9 ± 0.5	—

The experimental data reveal a smoother mass loss than suggested by the OEP description (eqn (5)). In a refined approach, a reduction of the evaporation surface (RES) needs to be considered becoming effective when the water content for a process has reached a sufficiently low level, corresponding, *e.g.*, to a monolayer. Below this transition temperature (*T*_trans_), the mass loss is only governed by thermodynamic parameters, whereas above *T*_trans_, it is assumed that the surface available for evaporation is proportional to the remaining mass of water of a specific type. The onset of the surface reduction may be smeared out characterized by a transition width *G* for various reasons, like the finite time needed for water to diffuse out of the sample or because of inhomogeneities in the water anchoring sites. The fits obtained in this way describe the data well (Fig. 5b), and the fit results are given in Table 1. It is noted that the transition widths *G* in the RES model of 8.8 K and 0.9 K are small compared to the corresponding values of the transition temperatures *T*_trans_ of 353.0 K and 422.8 K (Table 1).

An alternative approach yielding a description of almost equal quality is obtained by invoking a distribution of the activation energies (DAEs) of evaporation. Such a model originally suggested in 1961 to describe sorption isotherms was named “homotattic patch approximation” and describes the division of the total surface into small homogeneous areas with individual energies.^27^ In our case, the interfacial water molecules exist in different environments arising from the faceted nature of the IONPs as well as from inequivalent binding sites for water with different functional groups of the ligands or other water molecules. Essential fit results are presented in Table 1, too. Details for the definition of the models are given in the SI, Section 4.2, and the complete sets of fitting results are given in Tables S4 and S5 for various GD values of CIT and DEG.

It is to be noted that the three models, although invoking physically different processes, yield within errors the same values for the thermodynamic parameters *E*_*i*_ and *k*_*i*_ as well as for the mass fractions *s*_*i*_. However, further details of an evaporation process like the extent of multilayer formation become highlighted in the RES-description, which refers to a fundamental property of any desorption process. If appropriate, a possible smearing of the energy landscape of the binding sites is being called upon in the DEM model.

**Fig. 6:**
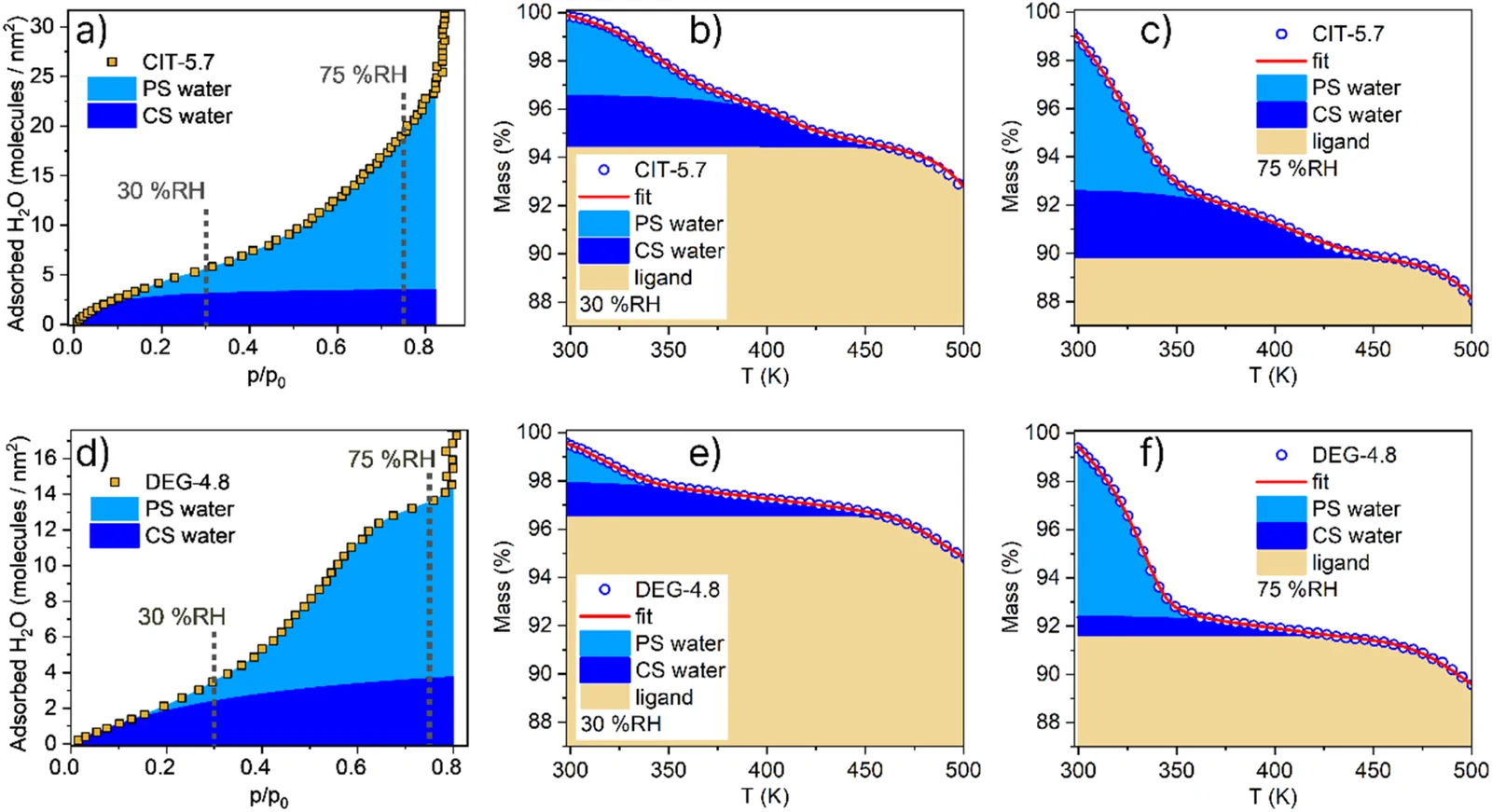
(a) Water sorption curve of CIT-5.7; vertical dotted lines indicate the RH of 30% and 75% used for TGA measurements in panels (b) and (c), respectively. Panels (d–f) show similarly for the DEG-4.8 sample. Dark blue and light blue fillings represent chemisorbed (CS) and physisorbed (PS) water in all panels.

The mass losses of water for CIT-5.7 and DEG-4.8 in Fig. 6 can be separated consistently for both humidities into two processes with evaporation energies of ∼32 kJ mol^−1^ (∼30 kJ mol^−1^ for CIT and ∼34 kJ mol^−1^ for DEG) and ∼41 kJ mol^−1^ (for both CIT and DEG). These values are within the usual ranges for physisorbed (PS) water of 15–30 kJ mol^−1^ and for hydrogen bond dissociation and chemisorbed (CS) water of 40–60 kJ mol^−1^, see TGA fit results in Table 2.^28,29^ This supports the interpretation that PS water represents molecules in the multilayer associated with ligands by van der Waals interactions or coordinated to other water molecules *via* hydrogen bonds. In contrast, CS water represents water molecules coordinated with ligand functional groups through hydrogen or covalent bonding. On bare metal oxide surfaces, chemisorption of water is often accompanied by water dissociation resulting in a hydroxylation of the metal surface, although non-dissociative chemical bonds exist as well.^5^ Since our IONPs do not feature any surface hydroxylation,^22^ and since all investigated CIT loadings are at or above the monolayer coverage leaving no significant IONP surface for water dissociation, we assume that the CS water binds to the ligand functional groups. In a previous quasielastic neutron scattering study, we found an activation energy of 18 ± 6 kJ mol^−1^ for the diffusion of interface water for relatively dry (8% RH) CIT-IONPs.^9^ This shows that the activation energy for water motion is approximately 10–20 kJ mol^−1^ lower than for water desorption.

**Table 2:** TGA fit parameters from RES model for water-related steps of CIT-5.7 and DEG-4.8 samples equilibrated at 30 and 75 RH% each. *k*_*i*_ are pre-exponential factors (eqn (5)), *E*_*i*_ denotes activation energies of the processes, *s*_*i*_ refers to the mass fractions, *T*_trans*i*_ and *G*_*i*_ are the temperature of transition of the reaction order from 0 to 1 and the corresponding temperature widths

Sample name	CIT-5.7 at 30% RH		CIT-5.7 at 75% RH		DEG-4.8 at 30% RH		DEG-4.8 at 75% RH	
	PS	CS	PS	CS	PS	CS	PS	CS
*k*_*i*_ × 10^−5^/s^−1^	1.16 ± 0.16	7.85 ± 0.59	3.86 ± 4.16	9.21 ± 0.10	11.54 ± 1.12	11.76 ± 5.23	19.55 ± 12.29	22.19 ± 12.88
*E*_*i*_/kJ mol^−1^	29.51 ± 0.33	42.22 ± 0.01	30.40 ± 2.75	40.21 ± 0.06	32.68 ± 0.18	39.59 ± 0.11	35.32 ± 1.66	42.55 ± 0.78
*s*_*i*_/wt%	3.51 ± 0.07	2.13 ± 0.12	6.47 ± 0.31	2.87 ± 0.30	1.73 ± 0.01	0.96 ± 0.06	7.08 ± 0.01	0.84 ± 0.07
*T*_trans*i*_/K	353.0 ± 0.9	422.8 ± 1.8	335.4 ± 3.1	326.7 ± 0.2	330.0 ± 19.1	359.4 ± 65.0	345.2 ± 1.2	390.0 ± 21.9
*G*_*i*_/K	8.8 ± 0.7	0.9 ± 0.5	12.8 ± 3.5	20.5 ± 1.8	48.4 ± 12.4	13.29 ± 7.7	5.1 ± 1.6	5.0 ± 11.9

The total amount of water scales well with the loading of water at different RHs as shown for CIT-5.7 equilibrated at RH 30% in Fig. 6b and at RH 75% in Fig. 6c, as well as for DEG-4.8 at the same RH values and shown in Fig. 6e and f. The amount of ligand is independent of the water loading in both cases. Furthermore, for both ligands the amount of CS water above a threshold value is constant underpinning the interpretation of CS water interacting with functional groups of the ligands. All functional groups are to a large extent saturated with CS water at 30% RH. In turn, the amount of PS water increases with the water loading, implying that water adsorbed beyond 30% RH interacts dominantly with the already adsorbed water (Table 3). The assignment of two different types of water is further supported by water vapor sorption measurements as evidenced in Fig. 6a for CIT-5.7 and in Fig. 6b for DEG-4.8. They first show a Langmuir-type sorption of CS water with a progressive filling of a first layer, followed by a transition to a build-up of multilayers of PS water for higher water loadings.

**Table 3:** Summary of sample composition for CIT-5.7 and DEG-4.8 at 30 and 75% RH each: water and ligand contents. PS and CS water fractions correspond to mass fractions of Table 2

Sample	CIT-5.7 at 30% RH	CIT-5.7 at 75% RH	DEG-4.8 at 30% RH	DEG-4.8 at 75% RH
Ligand/wt%	27 ± 0.1	27 ± 0.1	11.0 ± 0.1	10.0 ± 0.1
Water total/wt%	5.6 ± 0.1	9.4 ± 0.4	2.7 ± 0.1	7.9 ± 0.1
PS water/wt%	3.5 ± 0.1	6.5 ± 0.3	1.7 ± 0.01	7.1 ± 0.01
CS water/wt%	2.1 ± 0.1	2.9 ± 0.3	1.0 ± 0.1	0.8 ± 0.1

### 3.5 Ligand and water binding energies

**Fig. 7:**
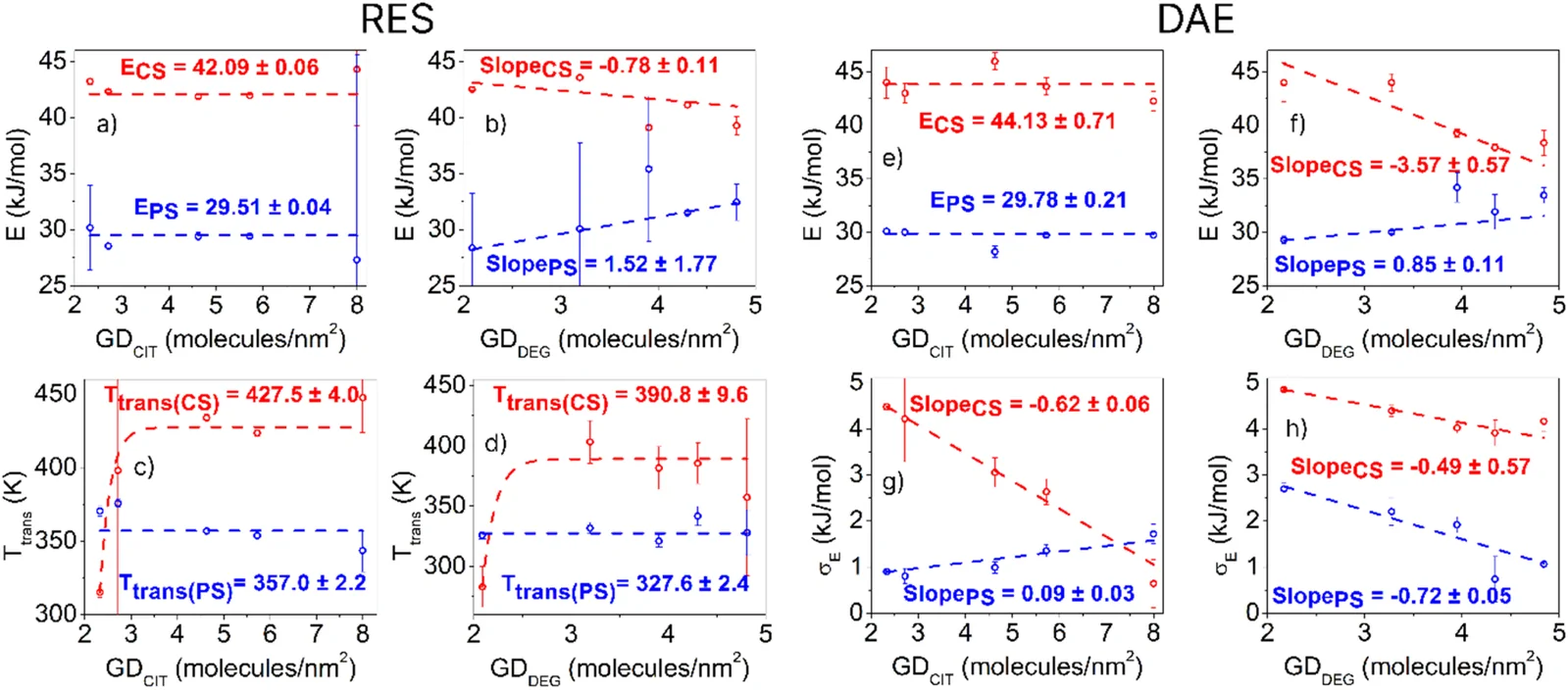
Evolution of essential fit parameters as a function of GD for CIT (a, c, e and g) and DEG (b, d, f and h), both at 30% RH with data points for PS water in blue and CS water in red. (a–d) RES model with activation energy E and transition temperature *T*_trans_; (e–h) DAE model with activation energy E and energy width *σ*_E_. See Tables S4 and S5 for full list of fitting parameters.

In the following, we discuss the interface properties as a function of ligand coverage from a ligand monolayer (samples CIT-2.3 and DEG-2.1) to 3.5 monolayers for CIT (CIT-8.1) and 2.3 monolayers for DEG (DEG-4.8). Fig. 7 shows the most relevant parameters activation energy and transition temperature for the RES model and activation energy and energy distribution for the DAE model. Tables S4 and S5 display the fit results for the RES and DAE models for samples with various CIT and DEG GDs.

Most importantly, the distinction between CS and PS water is maintained at all ligand coverages both for CIT and DEG (Fig. 7). In the case of CIT-covered samples, both models yield activation energies that remain constant at energy levels of about 30 kJ mol^−1^ and 42 kJ mol^−1^ for PS and CS, respectively.

The lower transition temperatures according to RES fitting for PS water of 357.0 ± 2.2 K for CIT and 327.6 ± 2.4 K for DEG samples are independent of the GD, while the transition temperatures for CS show saturation approaching a plateau once the GD exceeds the monolayer (427.5 ± 4.0 K for CIT and 390.8 ± 9.6 K for DEG, values obtained with an exponential fit). When the GD exceeds monolayer coverage, additional ligands provide a larger number of equivalent water-binding sites resulting in interfacial layers where water desorption occurs at higher temperatures.

In the DAE description for the CIT case, the energy distribution *σ*_E*i*_ for PS water increases slightly from 0.9 to 1.6 kJ mol^−1^ with increasing GD (Fig. 7g), while it diminishes more pronouncedly from 4.5 to 0.5 kJ mol^−1^ for CS water. This indicates that increasing the GD_CIT_ introduces additional binding sites for CS water. As the surface becomes saturated with ligands beyond the monolayer, these sites influence the desorption already at lower temperature. This shows that CS water interacts with the functional groups of the ligands. As the GD increases, the surface becomes populated with a higher number of chemically similar functional groups creating a more uniform local binding environment for CS water and thus providing a sharper desorption step in TGA.

In DEG-grafted samples, the activation energies of both CS and PS water exhibit only modest changes with GD, which reflects the distinct binding environments induced by DEG: the increase in *E*_PS_ (Fig. 7f) suggests that PS water interacts with a partially structured hydration layer near the DEG hydroxyls, while the decrease in *E*_CS_ indicates weaker and more uniform interactions with the flexible DEG ligands. The narrowing of *σ*_E*i*_ for both populations with increasing GD (Fig. 7h) is consistent with DEG creating a more homogeneous local environment.

Based on a combined analysis of TGA and water vapor sorption measurements, we propose a sequential water adsorption for CIT and DEG ligands on IONPs. At low humidity with *P*/*P*_0_ < 0.2, water molecules adsorb primarily onto ligand functional groups which are carboxylates in the case of CIT and hydroxyl and ether groups in the case of DEG, forming CS water. With increasing water inventory, the adsorption mechanism shifts from chemisorption to physisorption, and water–water interactions become more relevant, while at the same time the influence of the ligand diminishes. Specifically, in the case of CIT-5.7 sample at 30% RH, there are 1.2 ± 0.6 CS water molecules per CIT. At 75% RH, the amount of CS content is the same, while the amount of PS water rises to 3.0 ± 0.6 water molecules per CIT molecule. In the case of DEG the monolayer sample DEG-2.1 exhibits 1.2 ± 0.2 CS water molecules per DEG, *i.e.* identical to CIT within errors. This suggests that the initial CS regime depends primarily on the number of accessible high-affinity sites rather than on the number of polar functional groups. Cooperative hydrogen bonding and water sharing between neighbouring ligands may lead to a similar first-shell hydration stoichiometry for both ligand systems.

## 4. Conclusion

Vapor sorption and TGA experiments have been performed on CIT and diethylene glycol grafted iron oxide nanoparticle powders across a wide range of relative humidities and yielded consistent insight into the water sorption behaviour. Both techniques distinguish chemi- and physisorbed water species. While chemisorption on bare metal oxide surfaces is commonly understood to involve dissociation of water molecules and the formation of hydroxyl groups, here we interpret chemisorbed water as hydrogen bonded to functional (carboxyl and hydroxyl) groups of ligand molecules. This chemisorbed water is identified as the Langmuir adsorption step in the vapor sorption isotherms. For increasing relative humidities above about 10% RH in the case of CIT and 20% for DEG, physisorbed water is bound to ligands by van der Waals interactions or coordinated to other water molecules *via* hydrogen bonds and it appears to form multilayers.

In order to model the TGA data, two approaches yielded similar fit qualities: the RES model is based on the reduction of the evaporation surface towards the end of an evaporation step, and describes the evaporation process through the transition temperature and activation energy; in contrast, the DAE model parametrizes an activation energy and the width of its distribution, in order to address a distribution of activation energies which arises from the nature of different adsorption sites due to the complexity of the interface (facets, ligands, different functional groups). The main parameter being the activation energy is consistently identifying chemi- and physisorbed water for both fitting models across all grafting densities – of 2.3 to 4.8 and 8 wt% for DEG and CIT, respectively, and for all humidities from close to 0 up to 98% RH. The activation (desorption) energies of chemisorbed and physisorbed water fractions were determined to be 29–33 and 40–46 kJ mol^−1^, respectively, consistent for both models.

Importantly, the concept of cluster-to-film adsorption of water previously established for bare nonporous mineral surfaces can be transferred to describe vapor sorption isotherms and TGA data of ligand-grafted nanoparticle powders in this work. Hereby, for the first time to our knowledge, we demonstrate the applicability of this model to vapor sorption of ligand-grafted nanoparticles.

From the consistency of TGA and vapor sorption results follows that fitting TGA curves with the proposed RES or DAE model presents an insightful approach to characterize the fractions of chemi- and physisorbed water from TGA data alone with TGA being a widely available laboratory technique. Importantly, heating ramps in TGA were run with 5 K min^−1^ only for a proper separation of desorption steps.

These findings establish quantitative relationships between surface chemistry and hydration behavior, providing a foundation for controlling interfacial properties in nanomaterials and advancing fundamental understanding of water interaction with ligand-grafted IONPs.

## Author contributions

M. Z. conceived and supervised the project, contributed decisively to the scientific content and wrote essential parts of the manuscript, in particular for the final version. M. P. prepared the samples, carried out the experimental work (except TEM) and did the analysis of the results with substantial guidance by M. Z. and A. M. He wrote a preliminary draft of the manuscript. S. E. performed TEM measurements, and A. M. was strongly involved in the scientific interpretation of the data and wrote significant parts for various versions of the manuscript.

## Conflicts of interest

There are no conflicts to declare.

## Supplementary Material

NA-OLF-D6NA00573J-s001

## Data Availability

The data analysed for this article are available at https://rwth-aachen.sciebo.de/s/cM4n56PEqfsDyCj. Supplementary information (SI): sample characterization results (DLS, TEM, PXRD), calculation of ligand grafting densities, IR spectroscopy on citrate binding, TGA data, modelling and fit parameters. See DOI: https://doi.org/10.1039/d6na00573j.
