# Supplementary material for: Quantitative TGA distinguishing physisorbed and chemisorbed water in the ligand layer of iron oxide nanoparticles
Source: Nanoscale Adv. 2026 Aug 28. Online ahead of print. doi: 10.1039/d6na00573j (PMC13574204; doi:10.1039/d6na00573j)
Supplement: Supplementary file 1 [file NA-OLF-D6NA00573J-s001.pdf]

Supplementary Information (SI) for Nanoscale Advances.
This journal is © The Royal Society of Chemistry 2026

Supplementary Information for

Quantitative TGA Analysis Distinguishing Physisorbed and Chemisorbed Water in the Ligand Layer of Iron Oxide Nanoparticles

Mirijam Zobel[1,2]*, Maksim Plekhanov[1], Sascha Ehlert[4], Andreas Magerl[2,3]

[1]RWTH Aachen University, Institute of Crystallography, Jägerstrasse 17-19, 52066 Aachen, Germany

[2]Forschungszentrum Jülich GmbH, JCNS-3: Neutron Analytics for Energy Research, Wilhelm-Johnen-Straße, 52428 Jülich, Germany

[3]Forschungszentrum Jülich GmbH, JCNS-1: Neutron Scattering and Soft Matter, Wilhelm-Johnen-Straße, 52428 Jülich, Germany

[4]Friedrich-Alexander Universität, Biophysics Group, Department of Physics, Henkestraße 91, 91052 Erlangen, Germany

1. Sample characterization and geometrical considerations for nanoparticles………2

2. Binding of citrate to the IONPs surface………………………………..………...…………..5

3. Thermogravimetric analysis ………………………………………….…………….………7

1. Sample characterization and geometrical considerations for nanoparticles

1.1 Size and crystalline structure

Table S1 summarizes the 14 different samples synthesized and characterized in this work. The diameters of IONPs were determined by DLS for all samples and validated by PXD and TEM for selected samples. The number-weighted particle size distributions from DLS are shown in Fig. S1.

Table S1: Sample name with indicated ligand, grafting densities according to eq. (viii) (see chapter 1.2), number of additional washing cycles (performed with water for CIT and with ethanol for DEG), and diameters of IONPs measured with DLS, PXD, and TEM.

Sample name	Ligand grafting density, molecules/nm^2	Number of additional washing cycles	D_{DLS} / nm	D_{TEM} / nm	D_{PXRD} / nm
CIT-8.1	8.1 ± 0.8	0	8.2 ± 1.1		
CIT-8.0	8.0 ± 0.8	0	8.4 ± 1.3	5.7 ± 1.8	7.2 ± 0.2
CIT-5.7	5.7 ± 0.4	1	7.4 ± 1.1	6.5 ± 1.7	6.7 ± 0.1
CIT-4.8	4.8 ± 0.4	1	7.8 ± 1.2		
CIT-4.6	4.6 ± 0.4	1	7.1 ± 1.2		
CIT-2.7	2.7 ± 0.3	2	6.9 ± 1.0		
CIT-2.5	2.5 ± 0.2	2	7.8 ± 1.1		
CIT-2.3	2.3 ± 0.2	6	6.6 ± 1.2	5.3 ± 1.5	5.9 ± 0.1
DEG-4.9	4.9 ± 0.5	2	7.5 ± 1.1	6.7 ± 2.0	6.6 ± 0.1
DEG-4.8	4.8 ± 0.5	2	6.9 ± 1.1		
DEG-4.3	4.3 ± 0.5	3	7.7 ± 1.3		
DEG-3.9	3.9 ± 0.4	3	8.0 ± 1.4		
DEG-3.2	3.2 ± 0.3	4	8.1 ± 1.4	5.8 ± 1.3	7.2 ± 0.2
DEG-2.1	2.1 ± 0.2	4	7.4 ± 1.3	7.2 ± 0.1	5.3 ± 1.3

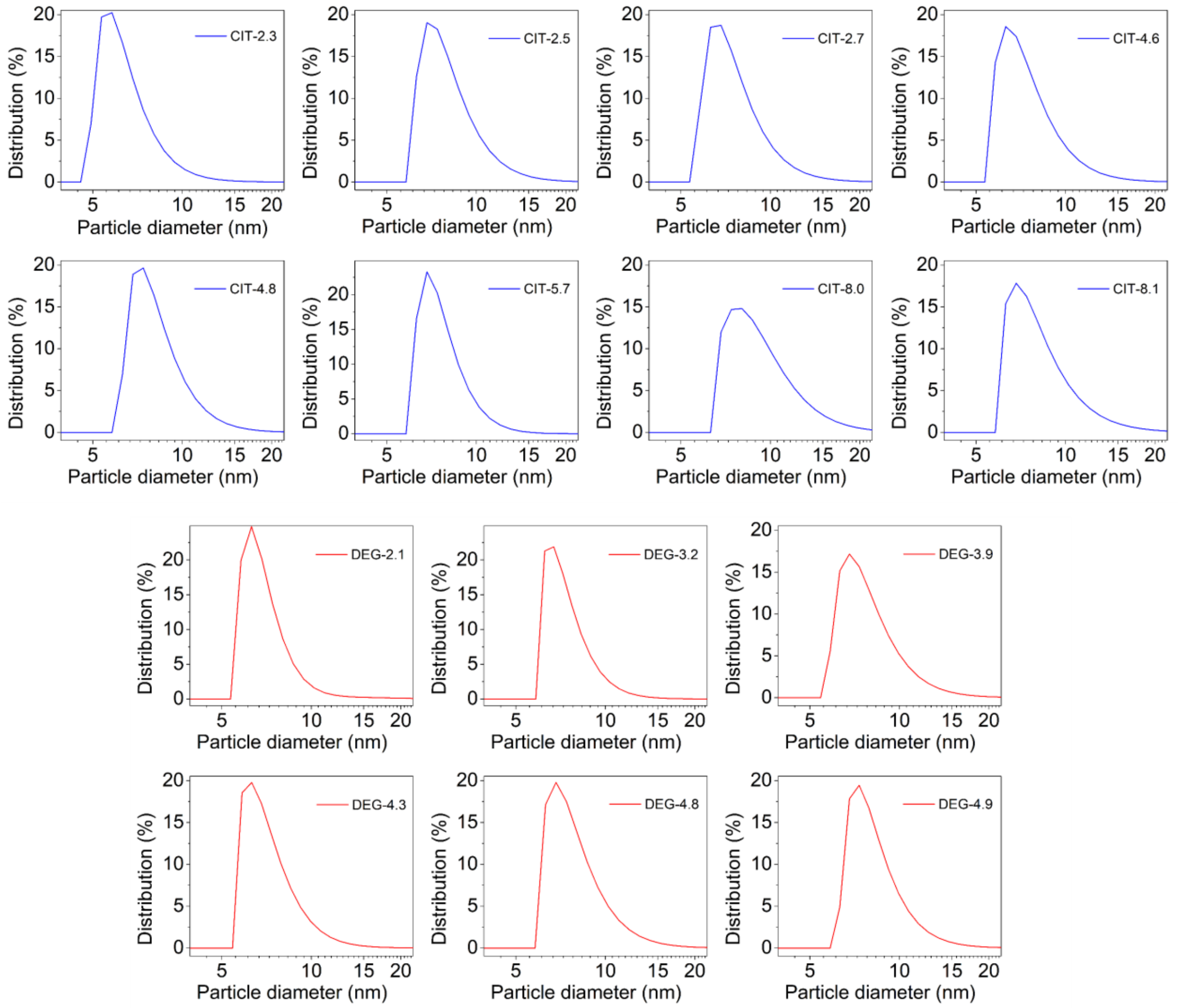

Fig. S1: Number-weighted particle size distributions obtained with DLS for all samples. Lognormal fit results are given in column 4 of Table S1.

According to TEM images shown in Fig. S2 for CIT-5.7 and DEG 3.2 as examples, all IONPs have a diameter between 5 and 7 nm, independent of the ligand (see Table S1).

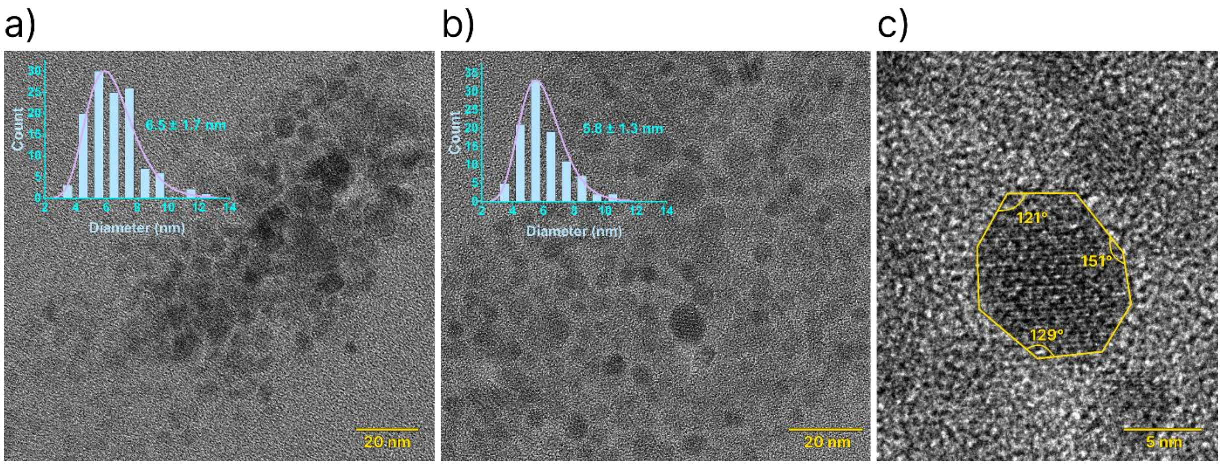

Fig. S2: TEM image of a) CIT-covered IONPs with diameters of 6.5 ± 1.7 nm and b) DEG-covered IONPs with diameters of 5.8 ± 1.3 nm. Insets show histograms together with a fitted lognormal distribution of the particle size. c) Enlarged IONP with outlined shape.

The magnetite nanoparticles exhibit a cube-octahedral or truncated octahedral (TO) morphology, as evidenced by the 8-edge polygonal projections observed in TEM images (Fig. S2). On the 2D projection of a particle, the interfacial angles between 121° and 151° are consistent with this geometry with angles near 120° corresponding to (111)-(111) facet interfaces, while angles between 129° and 151° represent (111)-(100) interfaces formed by the truncation of octahedral vertices. This morphology is in agreement with literature showing that (111) and (100) are the most stable surfaces of magnetite.[1] The TO geometry is to be considered as more realistic approximation for the shape of the IONP as compared to a spherical or octahedral geometry.

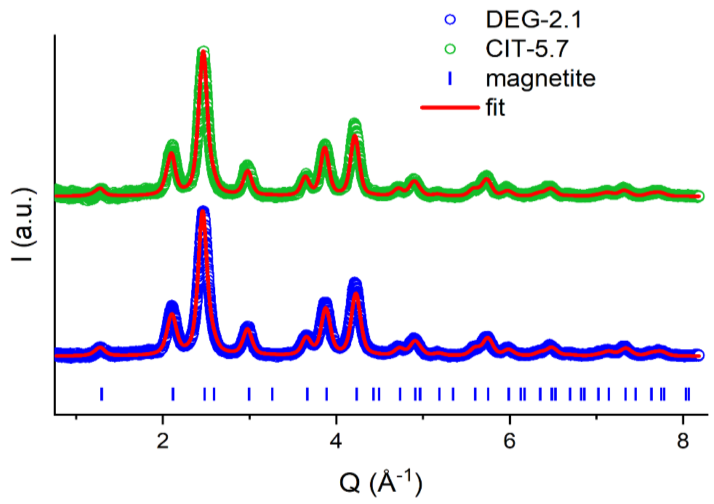

Fig. S3: Refined PXD patterns of DEG-2.1 and CIT-5.7 samples in the cubic (Fd-3m) magnetite structure.

The diameters of IONPs of DEG-2.1 and CIT-5.7 samples are 6.7 ± 0.1 and 7.0 ± 0.1 nm, respectively, according to Rietveld refinements of PXD patterns shown in Fig. S3. Further details of the refined parameters are given in Table S2. No significant difference is observed between CIT- and DEG-covered samples.

Table S2: Results of Rietveld refinements with a polynomial background fit of DEG-2.1 and CIT-5.7 samples in the cubic (Fd-3m) magnetite structure with lattice parameter a, isotropic thermal parameters for Fe, the particle diameter d, and the Rw factor.

Sample name	a, Å	U_{iso}Fe(8a)	U_{iso}Fe(16d)	d, nm	Rw, %
CIT-5.7	8.3896 ± 0.0004	0.0245 ± 0.0003	0.0170 ± 0. 0003	6.7 ± 0.1	9.4
DEG-2.1	8.3955 ± 0.0004	0.0246 ± 0.0003	0.0185 ± 0.0004	7.2 ± 0.1	8.8

The weighted R_w factor used as the goodness-of-fit criteria is defined as

$$R_w = \left[\frac{\sum_i^n w_i(y_i(obs)-y_i(calc))^2}{\sum_i^n w_i y_i(obs)^2}\right]^{0.5} * 100\% \quad \text{(i)}$$

with y_i(obs) and y_i(calc) being the observed and calculated intensities at the ith data point and w_i the error weight.

1.2 Ligand grafting densities

The wt.% of the ligands is obtained through the fitting of TGA data (see section 3). The grafting density is calculated for the TO geometry as outlined below. All edges of the NP are assumed to be of the same length, where the nanoparticle edge length L_{edge} is determined from the diameter of a circumscribed sphere *d* as

$$L_{edge} = \frac{d}{\sqrt{10}} \quad . \tag{ii}$$

The surface area of the TO IONPs is given by

$$A_{TO} = 6L_{edge}^2\left(1 + 2\sqrt{3}\right). \tag{iii}$$

In order to account for shape deviations from a regular TO, an additional shape error factor to the surface area (σ_{shape}) is calculated as

$$\sigma_{shape} = \frac{A_{octahedron} - A_{sphere}}{A_{TO}} \tag{iv}$$

where $A_{octahedron}$ and A_{sphere} are the surface areas of nanoparticles of ideal octahedral and spherical shapes of the same volume. From geometrical considerations it can be shown that these two values are related as $A_{octahedron} = 1.18 * A_{sphere}$. Assuming the A_{TO} as a midpoint between $A_{octahedron}$ and A_{sphere} the $A_{TO} = 1.09 * A_{sphere}$ is used in eq. (iv). This results in $\sigma_{shape} = \frac{0.18}{1.09} = 0.165$. With this, the total error of the surface area from the errors in size determination and shape is

$$\sigma_{surface} = \sqrt{\sigma_{shape}^2 + \sigma_{size}^2} \tag{v}$$

The volume of the TO IONPs is given by:

$$V_{TO} = 8L_{edge}^3\sqrt{2} \tag{vi}$$

The mass of a single TO IONP is identified through the density of magnetite ($\rho_{Fe_3O_4} = 5.17 g/cm^3$) and the volume of the NP according to $m_{NP} = \rho_{Fe_3O_4} \times V_{TO}$.

From the TGA data, the number of ligand or water per single NP and per unit of NP surface can be calculated. The number of ligands per nanoparticle ($N_{lig/NP}$) is calculated using their weight fractions ($\%wt$), molar mass of the ligand ($M_{CIT} = 189.12$ g/mol) and the Avogadro's number (N_A):

$$N_{lig/NP} = \frac{m_{NP} \times \%wt_{ligand} \times N_A}{\%wt_{NP} \times M_{CIT}} \tag{vii}$$

The grafting density is calculated in molecules/nm^2 as

$$GD_{lig} = \frac{n_{lig/NP}}{A_{NP(TO)}} \tag{viii}$$

To obtain the errors of the grafting densities (δ_D), the errors from TGA fitting and the DLS particle size are propagated through the eq. (ii) to (viii) by calculating the derivatives as:

$$\delta_D = \sqrt{\left(\frac{\partial D_{lig}}{\partial n_{lig/NP}} \delta_{n_{lig/NP}}\right)^2 + \left(\frac{\partial D_{lig}}{\partial A_{TO}} \delta_{A_{TO}}\right)^2} \tag{ix}$$

2. Binding of citrate to the IONPs surface

FT/IR measurements of samples with different grafting densities of CIT were performed (Fig. S4) to reach a better understanding of how the grafting density affects the coordination of the CIT molecules on the IONP surface. Four Gaussian peaks have to be fitted to the spectra in the wavenumber range of v = 1100 – 1800 cm^{-1} according to the eq. 2 in the main text (all parameters are refined). Peaks I (green) (1650 cm^{-1}) and II (orange) (1570 cm^{-1}) can be attributed to the asymmetric stretching $\nu_{as}(COO^{-})$ of the carboxylate groups in CIT, showing that at least two binding mechanisms for the carboxylate groups coexist. Peak III (1390 cm^{-1}) corresponds to the symmetric stretching $\nu_{s}(COO^{-})$ of carboxylate. These three peaks are related to the binding of CIT to the iron atoms of IONPs.[2] Peak IV (~1280 cm^{-1}) matches the most intense peak of the C-OH stretch related to the central hydroxyl group in CIT.[3] The wavenumber separation Δv between the symmetric stretch (peak III) and the area-weighted average of the peak positions of the asymmetric stretches (peaks I and II) decreases from 197.7 ± 2.3 to 192.9 ± 0.7 cm^{-1}. As can be seen from DFT-calculated spectra for mono- and bidentate binding mechanisms, the positions of symmetric and asymmetric stretching peaks are further apart for the monodentate binding. Thus, a decrease in the separation of peaks indicates that the monodentate fraction decreases and both oxygen atoms of a carboxyl group start to bind to the surface in either bidentate or bridging mode – both cases have a similar effect on the IR spectra as they are related to the number of oxygen atoms connected to the iron atom. The change in the binding mode allows for an increase in the grafting density of citrate.[2-4] When carboxylate groups transition from monodentate to bidentate coordination, the $\nu_{as}(COO^{-})$ stretch wavenumber increases more significantly than the $\nu_{s}(COO^{-})$ because the antisymmetric mode is more sensitive to changes in bond order differences between the C=O and C–O bonds.

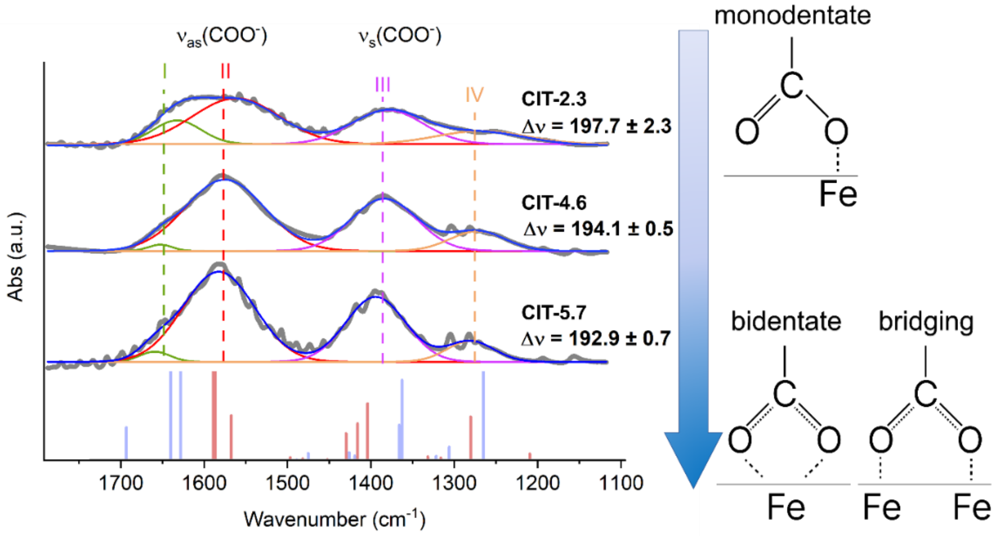

Fig. S4: FT/IR spectra of CIT-2.3, CIT-4.6, and CIT-5 samples. Green, red, purple, and orange curves correspond to Gaussian peaks I, II, III and IV, respectively. DFT calculated vibrational spectra at the bottom for monodentate (blue) and bidentate/bridging (red) binding modes are adapted from ref.[2].

The increase in monodentate binding at lower grafting density becomes possible as fewer ligands remain on the surface occupying a larger area and being positioned with two carboxylate anions closer to the surface and one carboxylate group being oriented away from the surface. In contrast, at higher ligand densities, the citrate molecules are forced to orient alongside the surface normal by binding through both oxygen atoms of a single carboxylate

group. The ratio of the area of peaks I and II shows that the contribution of the monodentate fraction decreases from CIT-2.3 to CIT-4.6 by 26 ± 8 %, while the monodentate fraction of CIT-4.6 and CIT-5.7 is about the same. This shows that CIT binds by the bidentate or bridging mode when the grafting density exceeds a monolayer coverage consistent with chelation binding via Fe atoms. This is illustrated in Fig. 2 of the main text.

The decrease in grafting density results in the broadening of the IR peaks as a larger number of molecular conformations as well as hydrogen bonds are available. Hydrogen bonds create a distribution of bonding environments of differing bonding strength. Additionally, hydrogen bonds are dynamic and constantly forming. This may result in the vibrational frequencies to fluctuate with time, which further broadens the IR absorption peaks.[5]

Table S3: Deconvolution of the IR spectra shown in Fig. S4. The parameters correspond to Gaussian functions defined in eq. 2 of the main text. ν_c is the wavenumber of the peak position, *w* is the peak width, and *A* is the peak area. The errors are obtained by fitting four Gaussian functions to the data.

Sample	Peak	ν_c / cm^{-1}	*w* / cm^{-1}	*A* / a.u.	(A_I/A_{II})*100 / %
CIT-2.3	I	1632.2 ± 1.1	57.5 ± 3.3	17.3 ± 3.9	28.6 ± 8.1
	II	1561.8 ± 2.4	104.2 ± 9.6	60.5 ± 10.3	
	III	1380.2 ± 1.3	85.7 ± 5.1	37.0 ± 6.6	
	IV	1267.5 ± 4.1	107.5 ± 19.1	17.9 ± 9.2	
CIT-4.6	I	1651.3 ± 1.0	28.6 ± 3.3	2.3 ± 0.4	2.7 ± 0.5
	II	1576.6 ± 0.4	95.5 ± 0.8	86.1 ± 0.7	
	III	1386.7 ± 0.3	78.7 ± 0.8	52.0 ± 0.5	
	IV	1272.1 ± 0.9	65.9 ± 1.8	16.4 ± 0.5	
CIT-5.7	I	1659.0 ± 2.1	36.4 ± 4.7	4.7 ± 0.9	4.8 ± 0.9
	II	1582.3 ± 0.5	85.9 ± 1.1	97.9 ± 1.3	
	III	1394.2 ± 0.4	71.4 ± 0.9	58.7 ± 0.7	
	IV	1280.5 ± 1.1	56.2 ± 2.5	14.8 ± 0.6	

3. Thermogravimetric analysis

3.1 Experimental results

TGA curves in Fig. S5 for both CIT and DEG covered samples before and after water sorption experiments show that the ligand content does not change. Note however, that the relative humidities for these measurements were not controlled and thus the water content may vary.

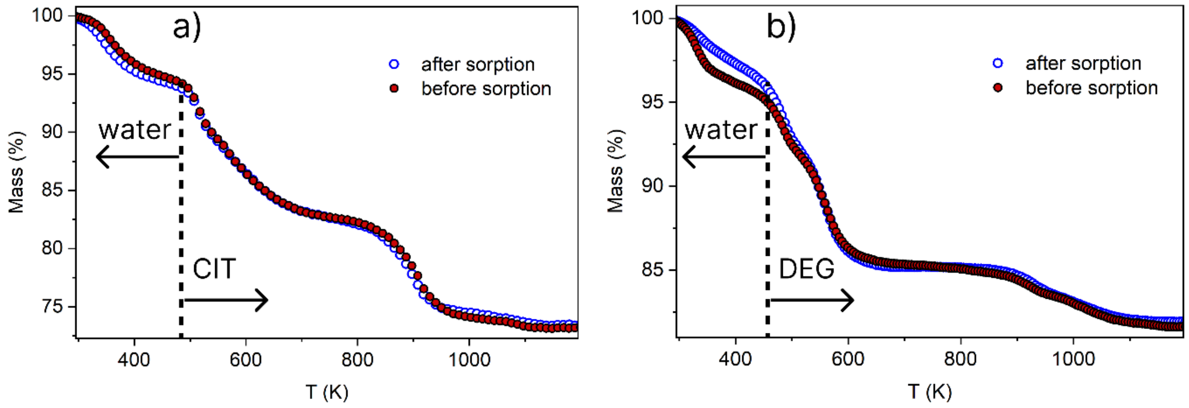

Fig. S5: TGA measurements of a) CIT-5.7 and b) DEG-4.8 before and after water sorption experiments as described in the experimental section of the main text show the stability of the ligand layer. Note that the water content is not controlled and may differ.

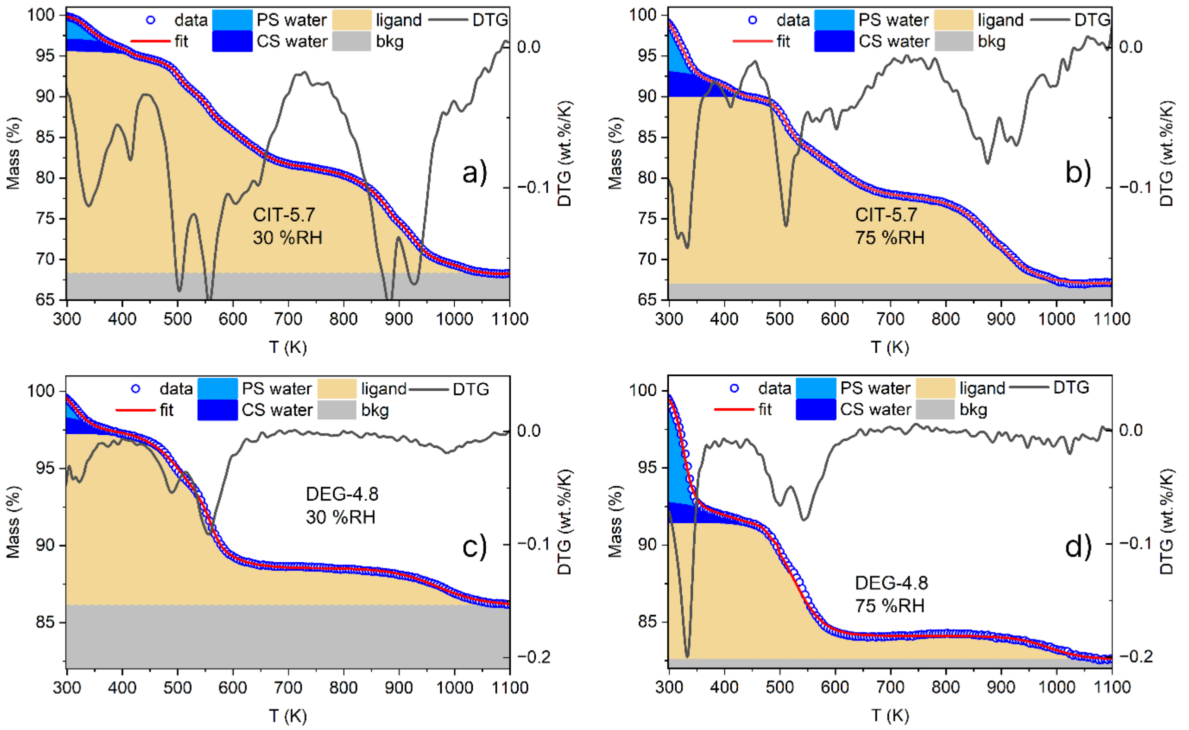

Fig. S6: TGA curves showing total mass loss of water and ligand for CIT-5.7 equilibrated at a) 30 %RH and b) 75 %RH, as well as for DEG-4.8 equilibrated again at c) 30 %RH and d) 75 %RH. PS and CS stand for physisorbed and chemisorbed water, respectively. DTG denotes the derivative thermogravimetric curve.

The separation of water and ligand contributions in the TGA mass loss is based on the CO_2 signal of the coupled MS measurement (Fig. S7).

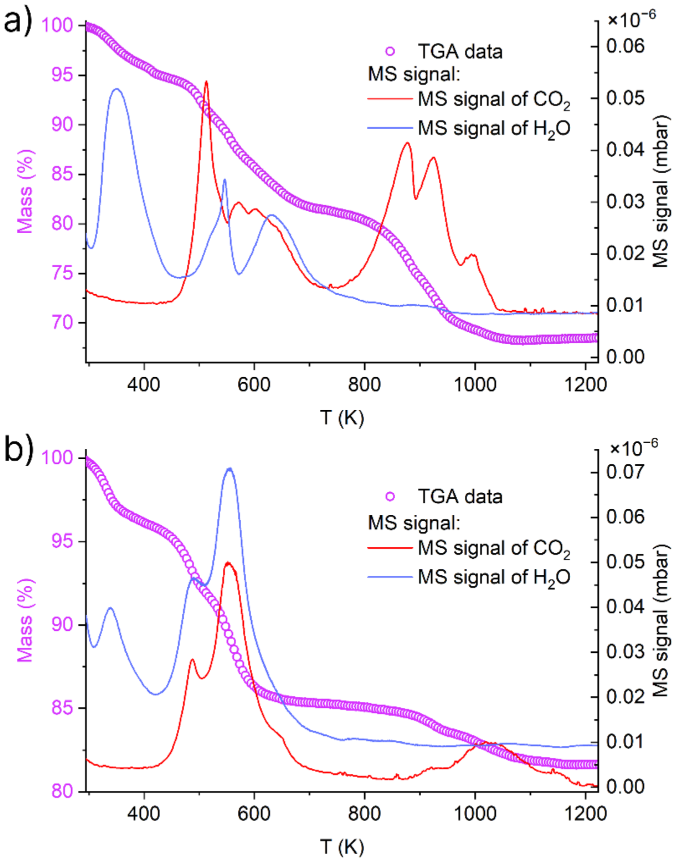

Fig. S7: Coupled TGA-MS measurements of a) CIT-5.7 and b) DEG-4.8. The MS signal of H_2O below the onset of the CO_2 signal stems from adsorbed water, whereas it originates from the decomposition of the ligand at higher temperatures.

3.2 TGA data modelling

The sample mass as a function of temperature in TGA measurements $m_{total}(T)$ is given by the following relation

$$m_{total}(T) = \sum_{i=1}^{p} m_i(T) + bkg \qquad \text{(x)}$$

where p is the number of desorption processes, $m_i(T)$ is the mass fraction from a process i, and bkg is the mass of the non-decomposing IONPs.

In a simple approach, the rate of mass loss for each process is given by the evaporation rate, which is assumed to follow an Arrhenius behavior according to eq. 5 and the remaining mass fractions are evaluated in Fig. 5a.

At a sufficiently low mass, e.g. when the adsorbate coverage becomes less than one monolayer for a process, the evaporation rate becomes in addition proportional to the remaining mass of the process i. This transition occurs at the temperature T_{trans_i}. To account for various reasons for inhomogeneities, like a finite diffusion time of vapor molecules out of the powder, the transition temperature may be smeared out, which can be mimicked with a sigmoidal function $n_i(T)$ with a temperature width G_i:

$$n_i(T) = 1/\left(1 + exp^{-G_i\left(T - T_{trans_i}\right)}\right) \quad \text{(xi)}$$

This model termed RES takes into account the reduction of the effective evaporation surface and needs to be applied towards the end of any evaporation process.

An alternative description considers the possibility of a distribution of the activation energies, termed DEA model. For each process, the remaining mass is given by

$$m_i(T) \ = \ s_i \int_{E_{min}}^{E_{max}} \theta_i(T, E) f_i(E) dE \quad \text{(xii)}$$

where s_i is a scaling parameter corresponding to the initial mass fraction of a process *i*. $f_i(E)$ is the activation energy distribution, defined with a Gaussian function

$$f_i(E) \ = \ \frac{1}{\sigma_{E_i}\sqrt{2\pi}} exp\left(-\frac{(E - E_i)^2}{\sigma_{E_i}^2}\right) \quad \text{(xiii)}$$

E_i is the central value of the activation energy of process *i* and σ_{E_i} is the standard deviation of its distribution. The integration limits from E_{min} to E_{max} in eq. xi are set to three standard deviations of the Gaussian width of the energy distribution σ_{E_i} in the present case.

The conversion function (θ_i) is implemented as a Polanyi-Wigner equation[6]

$$\frac{d\theta_i}{dT} \ = \ -\frac{k_i}{\beta} \theta_i^{n_i(T)} exp\left(-\frac{E_i}{RT}\right) \quad \text{(xiv)}$$

with the heating rate β, and a pre-exponential factor k_i, linked to the rate constant of evaporation at infinite temperature, and the universal gas constant $R = 8.3145\, Jmol^{-1}K^{-1}$. The parameter n_i corresponds to the order of the kinetics of the process *i*. In the case of n_i = 0, eq. (xiii) simplifies to the simple evaporation model OEP (main text, eq. 5), where no heterogeneity of desorption sites or geometric constraints are accounted for, as it can be expected for outer water layers. In this case, desorption will follow 0th order kinetics (n = 0), and the desorption rate depends only on the thermodynamic parameters (other factors like the geometry of the setup are not considered here).

We note that the RES description is fundamental and needs to be applied before the DAE-model is applied. This interpretation describes our data well. For completeness, Table S4 and Table S5 give a full account of fit results for both models. The most relevant parameters for the RES model E_i and T_{trans_i} are shown graphically in Fig. 7 of the main text for both CIT and DEG samples as a function of grafting density.

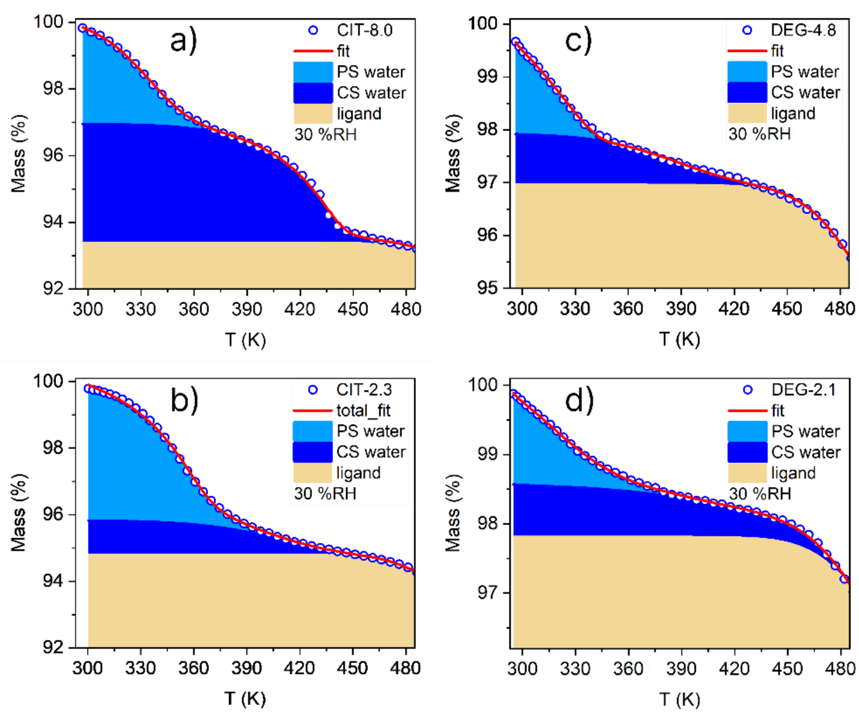

Fig. S8: Fitted TGA curves of CIT and DEG covered samples with different grafting densities each at 30 % RH: a) CIT-8.0, b) CIT-2.3, c) DEG-4.8 and d) DEG-2.1.

Table S4: TGA fit parameters for RES and DAE models for water-related steps for CIT-covered samples. E_i is the activation energy of a process, σ_{E_i} is the standard deviation of its distribution, s_i is the weight percentage, T_{trans_i} is the temperature of transition of the reaction order from 0 to 1, and G_i is the width of the temperature transition (RES).

Sample name		CIT-2.3 at 30%RH		CIT-2.7 at 30%RH		CIT-4.6 at 30%RH		CIT-5.7 at 30%RH		CIT-8.1 at 30%RH	
Water process		PS	CS	PS	CS	PS	CS	PS	CS	PS	CS
RES model	$k_i * 10^{-5}/s^{-1}$	1.14 ± 1.57	21.04 ± 10.74	0.91 ± 63.28	31.40 ± 30.69	1.07 ± 0.07	6.33 ± 0.67	1.16 ± 0.16	7.85 ± 0.59	1.06 ± 7.51	11.45 ± 16.36
	E_i / $kJ\ mol^{-1}$	30.20 ± 3.78	43.25 ± 0.35	28.55 ± 0.18	42.35 ± 0.142	29.47 ± 0.02	42.05 ± 0.12	29.51 ± 0.33	42.22 ± 0.01	27.42 ± 18.44	44.56 ± 5.02
	s_i / $wt.\%.$	4.08 ± 0.72	1.00 ± 0.68	3.09 ± 0.73	1.87 ± 0.90	2.76 ± 0.11	1.94 ± 0.13	3.51 ± 0.07	2.13 ± 0.12	2.94 ± 0.12	3.49 ± 0.10
	T_{trans_i} / K	370.4 ± 3.5	315.6 ± 3.0	375.8 ± 2.5	398.2 ± 207.2	83.7 ± 0.5	356.1 ± 2.5	353.0 ± 0.9	422.8 ± 1.8	343.1 ± 14.3	446.80 ± 23.8
	G_i / K	7.0 ± 5.1	5.1 ± 0.3	10.2 ± 54.1	14.1 ± 0.24	5.3 ± 0.1	4.8 ± 3.2	8.8 ± 0.7	0.9 ± 0.5	12.5 ± 25.3	5.0 ± 2.6
DAE model	$k_i * 10^{-5}/s^{-1}$	1.10 ± 0.04	15.10 ± 7.60	0.76 ± 0.20	25.68 ± 40.27	0.80 ± 0.17	26.71 ± 5.99	1.19 ± 0.25	9.39 ± 2.22	2.15 ± 0.19	5.96 ± 1.86
	E_i / $kJ\ mol^{-1}$	30.11 ± 0.02	43.97 ± 1.46	30.05 ± 0.16	42.96 ± 0.93	28.24 ± 0.52	46.01 ± 0.78	29.26 ± 0.36	41.72 ± 0.61	29.73 ± 0.32	42.28 ± 0.91

	σ_{E_i} / $kJ\ mol^{-1}$	0.90 ± 0.02	4.45 ± 0.08	0.74 ± 0.18	4.19 ± 0.09	0.93 ± 0.13	2.98 ± 0.32	1.14 ± 0.18	2.73 ± 0.53	1.59 ± 0.21	0.52 ± 0.52
	s_i / $wt.\%.$	3.79 ± 0.21	1.61 ± 0.16	3.66 ± 0.69	1.34 ± 0.62	2.10 ± 0.12	2.57 ± 0.11	2.65 ± 0.33	2.84 ± 0.36	2.88 ± 0.11	3.38 ± 0.11

Table S5: The same parameters as in Table S4 for DEG-covered samples.

Sample name		DEG-2.1 at 30%RH		DEG-3.2 at 30%RH		DEG-3.9 at 30%RH		DEG-4.3 at 30%RH		DEG-4.8 at 30%RH	
Water process		PS	CS	PS	CS	PS	CS	PS	CS	PS	CS
RES model	$k_i * 10^{-5}$ / s^{-1}	2.71 ± 2.39	15.51 ± 7.10	2.37 ± 7.10	9.59 ± 2.87	31.16 ± 80.42	6.79 ± 2.12	11.54 ± 1.12	11.76 ± 5.23	11.54 ± 1.12	11.76 ± 5.23
	E_i / $kJ\ mol^{-1}$	28.37 ± 4.86	42.50 ± 0.16	30.12 ± 7.79	43.79 ± 0.10	35.59 ± 6.44	39.31 ± 0.27	31.6 ± 0.14	41.47 ± 0.27	32.68 ± 0.18	39.59 ± 0.11
	s_i / $wt.\%.$	1.42 ± 0.23	0.93 ± 8.95	1.26 ± 0.06	0.86 ± 0.09	1.60 ± 0.15	1.35 ± 0.13	1.74 ± 0.09	0.95 ± 0.05	1.73 ± 0.01	0.96 ± 0.01
	T_{trans_i} / K	326.2 ± 2.5	402.8 ± 41.5	332.3 ± 4.8	404.3 ± 17.9	323.2 ± 4.2	383.1 ± 17.8	343.7 ± 7.9	387.9 ± 16.6	330.0 ± 19.1	359.4 ± 65.0
	G_i / K	5.0 ± 1.8	40.0 ± 39.9	5.0 ± 3.0	5.0 ± 17.0	5.0 ± 2.5	7.6 ± 6.5	49.4 ± 12.4	10.3 ± 7.7	48.4 ± 12.4	13.29 ± 7.7
DAE model	$k_i * 10^{-5}$ / s^{-1}	1.51 ± 0.18	2.53 ± 6.86	2.07 ± 0.30	8.27 ± 6.44	18.32 ± 8.77	6.97 ± 1.33	2.37 ± 1.52	9.77 ± 3.60	11.93 ± 3.49	4.16 ± 0.92
	E_i / $kJ\ mol^{-1}$	29.27 ± 0.10	43.95 ± 1.88	30.06 ± 0.08	44.01 ± 0.84	34.25 ± 1.31	39.31 ± 0.29	31.86 ± 1.76	37.92 ± 0.40	33.45 ± 0.83	38.36 ± 0.28
	σ_{E_i} / $kJ\ mol^{-1}$	2.68 ± 0.13	4.83 ± 0.03	2.07 ± 0.30	4.25 ± 0.13	1.71 ± 0.18	3.81 ± 0.12	0.49 ± 0.50	3.65 ± 0.29	1.60 ± 0.12	3.28 ± 0.22
	s_i / $wt.\%.$	1.47 ± 0.09	0.72 ± 0.95	1.14 ± 0.14	1.03 ± 0.24	1.25 ± 0.10	1.75 ± 0.10	2.64 ± 1.11	3.77 ± 1.19	1.78 ± 0.01	1.01 ± 0.01

References

1. D. Santos-Carballal, A. Roldan, R. Grau-Crespo and N. H. de Leeuw, *Phys Chem Chem Phys*, 2014, **16**, 21082-21097.
2. M. R. Noerpel and J. J. Lenhart, *J. Colloid Interface Sci.*, 2015, **460**, 36-46.
3. B. Smith, *Infrared Spectral Interpretation - A Systematic Approach*, Taylor&Francis Group, CRC Press LLC, United States of America, 1999.
4. M. Nara, H. Torii and M. Tasumi, *J. Phys. Chem.*, 1996, **100**, 19812-19817.
5. S. Bratos and H. Ratajczak, *J. Chem. Phys.*, 1982, **76**, 77-85.
6. C. T. Campbell and J. R. Sellers, *Chem. Rev.*, 2013, **113**, 4106-4135.
